# Lactate attenuates astrocytic inflammation by inhibiting ubiquitination and degradation of *NDRG2* under oxygen–glucose deprivation conditions

**DOI:** 10.1186/s12974-022-02678-6

**Published:** 2022-12-26

**Authors:** Jinying Xu, Tong Ji, Guichen Li, Haiying Zhang, Yangyang Zheng, Meiying Li, Jie Ma, Yulin Li, Guangfan Chi

**Affiliations:** 1 The Key Laboratory of Pathobiology, Ministry of Education, College of Basic Medical Sciences, Jilin University, Changchun, 130021 Jilin People’s Republic of China; 2 Department of Burn Surgery, The First Hospital of Jilin University, Changchun, 130021 Jilin People’s Republic of China; 3 Department of Pathogenic Biology, College of Basic Medical Sciences, Jilin University, Changchun, 130021 Jilin People’s Republic of China; 4 Department of Neurology, The First Hospital of Jilin University, Changchun, 130021 Jilin People’s Republic of China; 5 Department of Pathology, School of Basic Medicine, Qingdao University, Qingdao, 266071 Shandong People’s Republic of China; 6 School of Pharmaceutical Sciences, Jilin University, Changchun, 130021 Jilin People’s Republic of China

**Keywords:** Lactate, Inflammation, Astrocyte, *NDRG2*, Cerebral ischemia, Oxygen–glucose deprivation

## Abstract

**Background:**

Brain lactate concentrations are enhanced in response to cerebral ischemia and promote the formation of reactive astrocytes, which are major components of the neuroinflammatory response and functional recovery, following cerebral ischemia. *NDRG2* is upregulated during reactive astrocyte formation. However, its regulation and function are unclear. We studied the relationship between lactate and *NDRG2* in astrocytes under conditions of ischemia or oxygen–glucose deprivation (OGD).

**Methods:**

We examined astrocytic NDRG2 expression after middle cerebral artery occlusion (MCAO) using western blot and immunofluorescence staining. Under hypoxia conditions, we added exogenous L-lactate sodium (lactate) to cultured primary astrocytes to explore the effects of lactate on the ubiquitination modification of NDRG2. We profiled the transcriptomic features of NDRG2 silencing in astrocytes after 8 h of OGD conditions as well as exogenous lactate treatment by performing RNA-seq. Finally, we evaluated the molecular mechanisms of NDRG2 in regulating TNFα under OGD conditions using western blot and immunohistochemistry.

**Results:**

Reactive astrocytes strongly expressed *NDRG2* in a rat model of MCAO. We also showed that lactate stabilizes astrocytic *NDRG2* by inhibiting its ubiquitination. *NDRG2* inhibition in astrocytes increased inflammation and upregulated immune-associated genes and signaling pathways. *NDRG2* knockdown induced TNFα expression and secretion via c-Jun phosphorylation.

**Conclusions:**

We revealed that under OGD conditions, lactate plays an important anti-inflammatory role and inhibits TNFα expression by stabilizing *NDRG2*, which is beneficial for neurological functional recovery. *NDRG2* may be a new therapeutic target for cerebral ischemia.

**Supplementary Information:**

The online version contains supplementary material available at 10.1186/s12974-022-02678-6.

## Background

Neuroinflammation caused by ischemic stroke comprises a pathogenic process, leading to a worsened local neurological dysfunction and metabolic disorders. This neuroinflammation may represent a therapeutic target in terms of ameliorating the effects of ischemic stroke [1, 2]. Astrocytes, the most abundant cell type in the CNS, are key components of the neuroinflammatory response in concert with microglia [3]. Activated astrocytes differentiate into reactive astrocytes in a process known as reactive astrogliosis, a ubiquitous but poorly understood hallmark of all CNS pathologies, including ischemic stroke. Reactive astrogliosis is characterized by the upregulation of glial fibrillary acidic protein (GFAP) and cellular hypertrophy [4, 5]. Reactive astrocytes can produce either harmful or beneficial effects during CNS recovery following ischemic stroke, depending on their polarization subtype: anti-inflammatory phenotype or pro-inflammatory phenotype [5, 6]. A better understanding of the molecular mechanisms underlying the transition between different phenotypes in reactive astrocytes may contribute to the development of novel therapies for improving neurological function after ischemic stroke.

In the pathological conditions underlying ischemic stroke, a severe delay exists in the oxygen and glucose delivery to the injured portion of the brain. Lactate produced through the breakdown of glucose and glycogen stored intracellularly in astrocytes accumulates in the surrounding tissues [7]. Our previous research demonstrated that the tissue lactate content on the ipsilateral side in a rat model with middle cerebral artery occlusion (MCAO) was significantly increased during the early phases and that this increased lactate content acted on astrocytes, thereby causing astrogliosis and inducing a meaningful increase in axon guidance function [8]. Moreover, several recent studies have similarly reported that, under hypoxic conditions, lactate acts as a signaling molecule that plays an inhibitory role in the inflammatory response by modulating polarization and transcription in macrophages [9, 10]. However, the regulation of lactate in the neuroinflammatory response and immune regulation under conditions of cerebral ischemia has not been reported to date.

The *NDRG* (*N-Myc downstream-regulated gene*) family is one of the downstream regulatory genes of N-Myc (i.e., the N-Myc proto-oncogene protein), consisting of four family members (*NDRG1*, *NDRG2*, *NDRG3*, and *NDRG4*). All these members are highly conserved in structure, containing 57–65% of the same amino acid sequences [11]. Among them, *NDRG2* is specifically expressed in astrocytes and plays an important role in the physiological and pathological processes in the CNS [12–16]. Studies have shown that *NDRG2* plays an important role in regulating the polarization and inflammatory response in tumor-associated macrophages (TAMs). A lack of *NDRG2* in TAMs promotes M1 polarization, producing IL1, IL12, and TNFα, thereby inhibiting the tumor growth [17, 18]. This result suggests that *NDRG2* may function as an important regulator in cells with immunomodulatory functions, such as astrocytes.

In this study, we employed a model of a rat with MCAO and found that the *NDRG2* expression increased along with reactive astrogliosis. Moreover, we showed that the presence of lactate maintained the astrocytic *NDRG2* stability by inhibiting its ubiquitination. Genetic inhibition of *NDRG2* in astrocytes resulted in a strong increase in inflammation, upregulation of immune-related genes, and promotion of signaling pathways in vitro, with TNFα as the core. Additionally, we showed that the *NDRG2* knockdown induced the TNFα expression and secretion by increasing c-Jun phosphorylation. Hence, this study provides new insights into targeting *NDRG2* as a potential therapeutic and neuroinflammatory strategy for ameliorating the adverse effects of ischemic stroke.

## Results

### *NDRG2* in reactive astrocytes was upregulated in ischemia

**Fig. 1:**
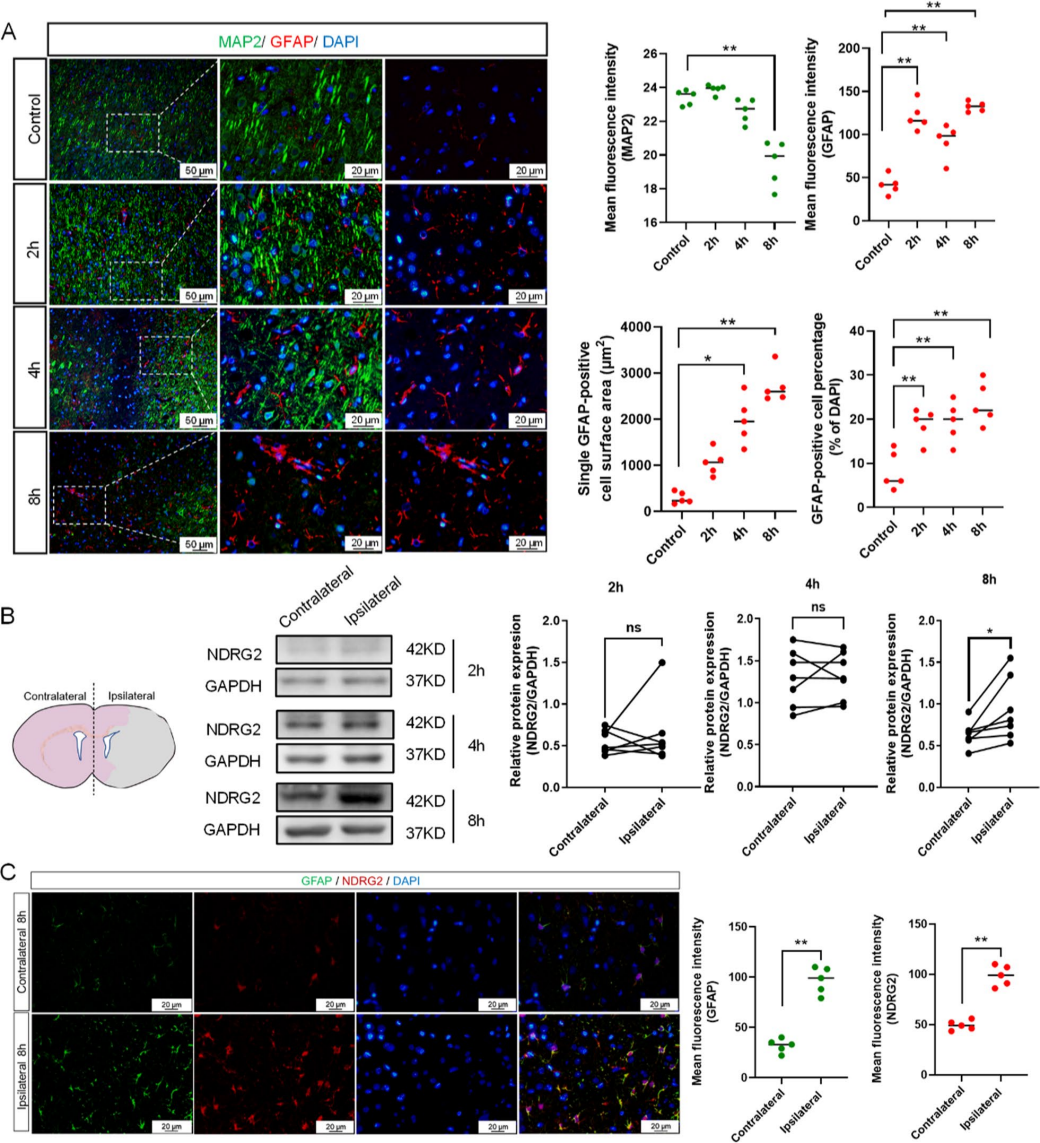
*NDRG2* levels in reactive astrocytes are upregulated in ischemia. **A** Representative images of the cortex after 2 h, 4 h, and 8 h of middle cerebral artery occlusion (MCAO), double-stained for glial fibrillary acidic protein (GFAP, red) and microtubule-associated protein 2 (MAP2, green). Scale bar: 50 μm or 20 μm. Statistical significance was assessed using one‐way ANOVA followed by ordinary ANOVA or nonparametric test. Data are expressed as means ± standard deviations (SD); *n* = 5 random samples, *P < 0.05 and **P < 0.01 vs. Control. **B** Western blot analysis of *NDRG2* expression in contralateral and ipsilateral brain tissues obtained 2 h, 4 h, and 8 h after MCAO surgery. Glyceraldehyde-3-phosphate dehydrogenase (GAPDH) is used as a loading control, with semi-quantification of western blot findings representing *NDRG2* expression levels. Paired t-tests were used for statistical comparisons. Data are expressed as means ± SD. *n* = 7, ns = no significance and *P < 0.05 vs. Contralateral. **C** Representative brain sections obtained 8 h after MCAO surgery, double-stained for GFAP (green) and *NDRG2* (red). Scale bar: 20 μm. Quantification of *NDRG2* and GFAP expression is shown in the right panel. Unpaired *t*-tests are used for statistical comparisons. Data are presented as means ± SD. *n* = 5 random samples, **P < 0.01 vs. Contralateral

We examined the *NDRG2* expression after MCAO in rat models. Double-staining immunofluorescence results showed that microtubule-associated protein 2 (MAP2) positive neurons decreased in number and that the MAP2 negative zone expanded over time in the ipsilateral zone, while GFAP-positive astrocytes showed hypertrophy and the GFAP expression was upregulated in the same zone (Fig. 1A). Western blot results showed that the increase in the *NDRG2* protein expression was statistically significant in the ipsilateral zone 8 h after MCAO (Fig. 1B). Immunofluorescence staining showed that the GFAP and *NDRG2* expressions were colocalized and the expression levels of both proteins were upregulated in the ipsilateral zone (vs. the contralateral zone) (Fig. 1C). These results suggest that the *NDRG2* expression was upregulated in reactive astrocytes in the ipsilateral zone within an MCAO model.

### Lactate upregulated *NDRG2* expression by suppressing ubiquitin modification

**Fig. 2:**
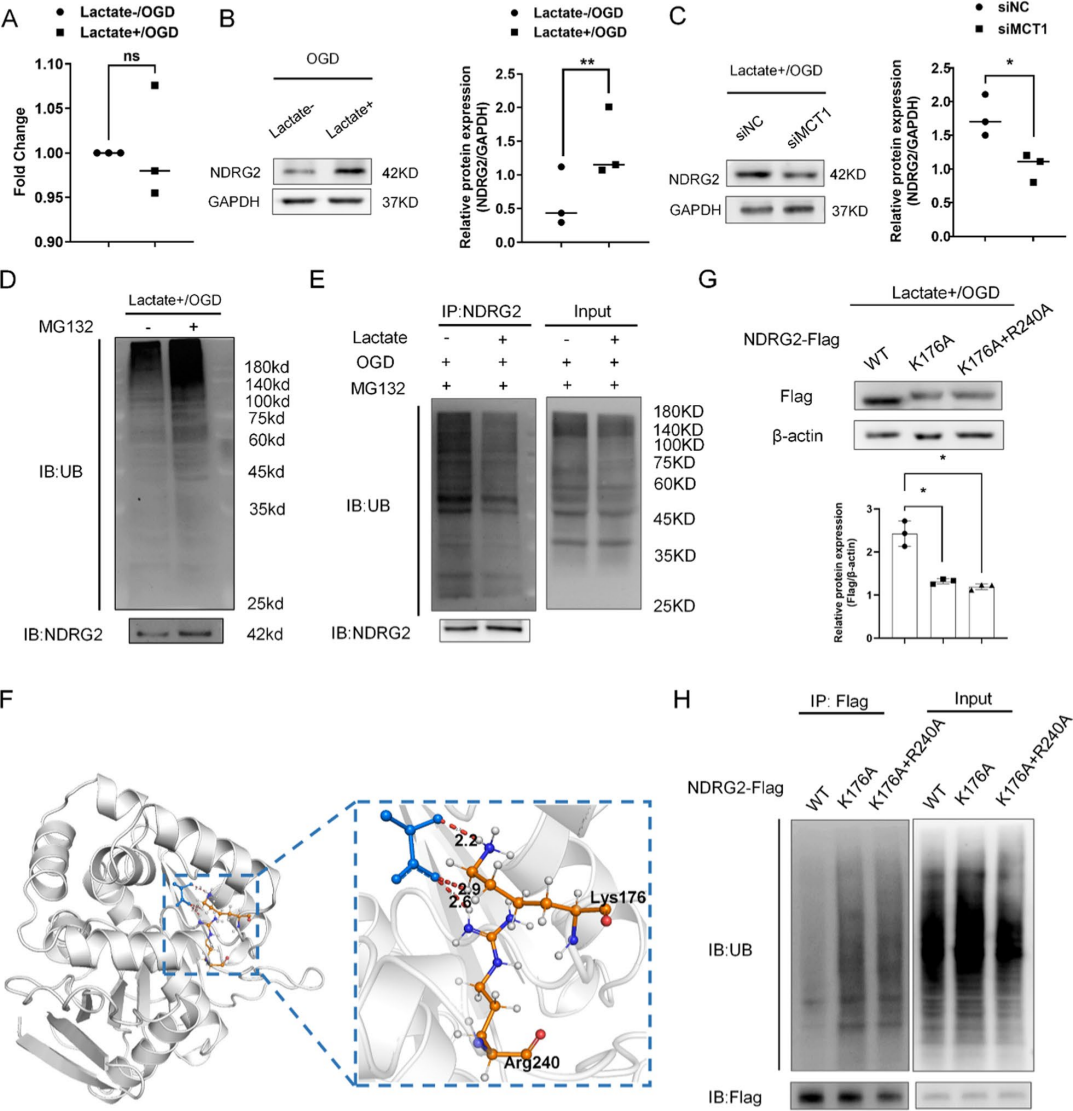
Under oxygen–glucose deprivation (OGD), extracellular lactate enters cells and upregulates *NDRG2* protein expression by suppressing its ubiquitination. **A** A qRT–PCR analysis of *NDRG2* mRNA after lactate treatment. GAPDH was used as an endogenous control. Paired t-tests were used for statistical comparisons. Data are expressed as means ± SD. *n* = 3, ns = no significance vs. Lactate-/OGD. **B** Western blot for the determination of *NDRG2* protein expression levels in ‘‘lactate−” and ‘‘lactate + ” groups. GAPDH was used as a loading control. Semi-quantification of western blot findings shows the relative protein expression. Paired t-tests were used for statistical comparisons. Data are expressed as means ± SD. n = 3, **P < 0.01 vs. Lactate-/OGD. **C** Western blot findings for *NDRG2* protein expression after siMCT1 transfection. GAPDH was used as a loading control. Paired t-tests were used for statistical comparisons. Data are expressed as means ± SD. n = 3, *P < 0.05 vs. siNC. **D** Western blot for determining the poly-ubiquitination of total proteins (UB) and *NDRG2*. **E** Ubiquitination assay of *NDRG2* in astrocytes in the ‘‘lactate−” and ‘‘lactate + ” groups. *NDRG2* was used as a loading control. **F** Molecular docking simulation studies were used to investigate the potential interactions between lactate and *NDRG2*. **G** Western blot to determine the two *NDRG2* variants mutated at the putative interaction sites (K176A and R240A) in astrocytes after applying OGD conditions and lactate stimulation. Statistical significance was assessed using one‐way ANOVA. Data are expressed as means ± SD; *n* = 3, *P < 0.05 vs. wild type (WT). **H** Ubiquitination assay of variant *NDRG2* proteins in astrocytes after applying MG132 and lactate stimulation under OGD conditions

Our previous study showed that lactate concentrations on the ipsilateral side increased after ischemic stroke [8]. To investigate the regulatory mechanisms specific to lactate and *NDRG2* under hypoxic conditions, we added exogenous L-lactate sodium (lactate) to cultured primary astrocytes. Since the expression of HIF-1α transcriptionally regulates the expression of *NDRG2* [19, 20], we investigated the possibility that HIF-1α regulates *NDRG2* protein expression under oxygen–glucose deprivation (OGD) conditions. The results showed that HIF-1α levels were not affected by lactate treatment (Additional file 1: Fig. S1). Similarly, the real**-**time quantitative reverse transcription polymerase chain reaction (qRT-PCR) analysis showed that lactate did not influence *NDRG2* mRNA expression (Fig. 2A). In contrast, western blot results showed that lactate upregulated the *NDRG2* protein levels (Fig. 2B). Interestingly, when monocarboxylate transporter (MCT1), a transporter mainly responsible for lactate uptake) was blocked by siRNA, the *NDRG2* levels decreased under exogenous lactate treatment (Fig. 2C). This finding indicates that extracellular lactate entered the astrocytes and upregulated the *NDRG2* levels via a translational or post-translational regulatory mechanism.

To additionally explore the effects of lactate on the ubiquitination modification of *NDRG2* under OGD conditions, we examined the ubiquitination level of *NDRG2* after treatment with MG132 (carbobenzoxy-Leu-Leu-leucinal; a proteasome inhibitor). Western blot results showed that the total ubiquitin level increased after treatment with MG132, as did the *NDRG2* levels (Fig. 2D). Under treatment with MG132, the ubiquitinated *NDRG2* expression decreased after exogenous lactate treatment (Fig. 2E).

Molecular docking simulation studies were used to investigate the potential interactions between lactate and *NDRG2*. Among the three best docking models (i.e., with docking results of 10^–3.1^ kcal/mol), we found that Lys176 formed hydrogen bonds with lactate in all three docking models. The Arg240-lactate hydrogen bonds were discovered in two models, indicating the critical importance of Lys176 in *NDRG2*-lactate binding. Additionally, we noted that Arg240 may be the second most important key amino acid residue (Fig. 2F). Western blot results showed that the site-directed mutagenesis of Lys176 significantly decreased the *NDRG2* levels compared to the wild-type *NDRG2* levels. In contrast, Lys176 and Arg240 double-site mutagenesis did not cause any additional decrease in *NDRG2* levels (Fig. 2G). Moreover, a ubiquitination assay showed that ubiquitin combined with *NDRG2* increased after Lys176 site-directed mutagenesis and Lys176 and Arg240 double-site mutagenesis (Fig. 2H). These findings indicate that, under OGD conditions, lactate upregulates *NDRG2* levels by suppressing the ubiquitin modification of *NDRG2*. We concluded that Lys176 is the key amino acid residue mediating this interaction.

### *NDRG2* deletion upregulated pro-inflammatory genes and pathways under OGD conditions

**Fig. 3:**
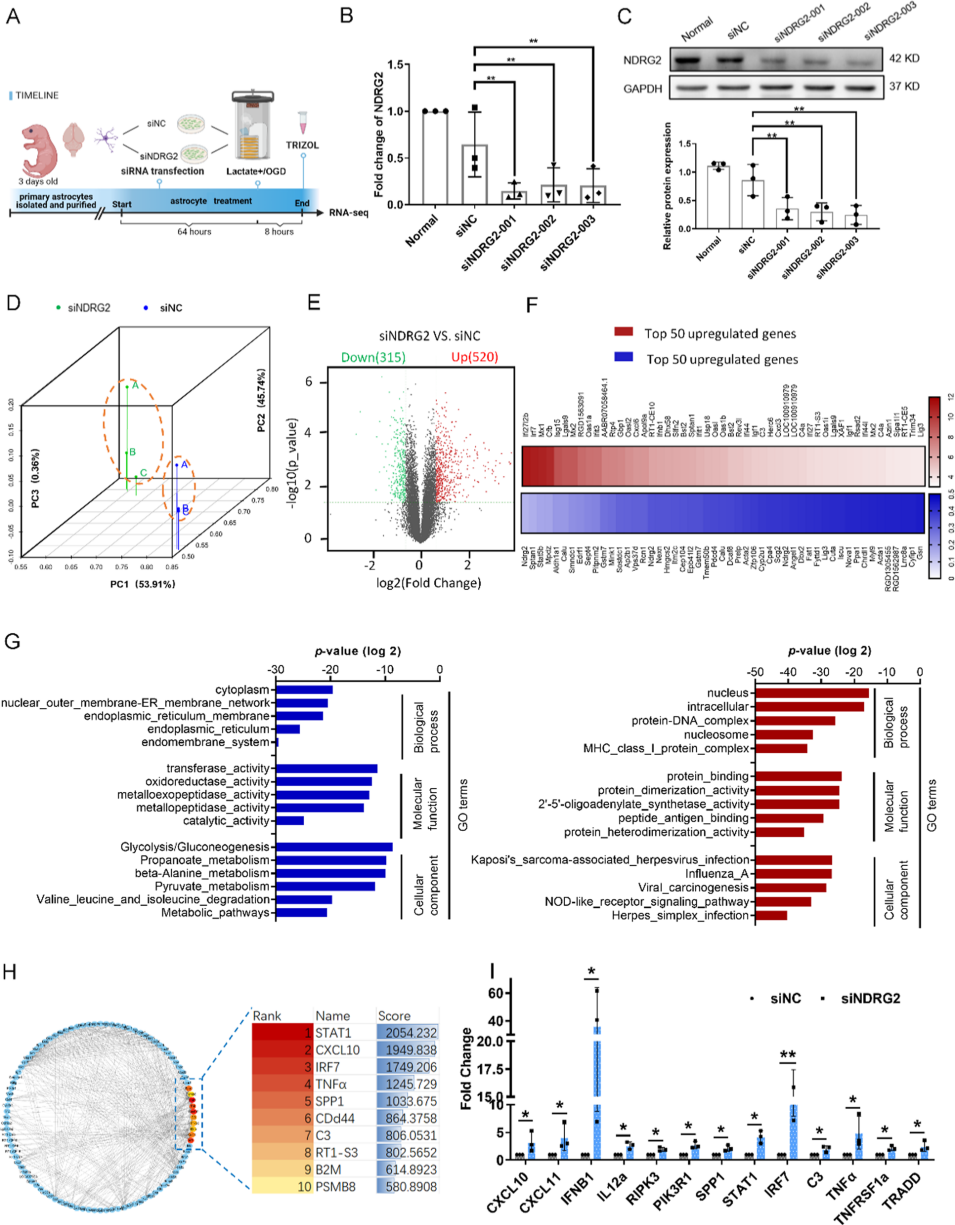
RNA-seq profiling of *NDRG2*-silenced astrocytes exposed to OGD conditions. *NDRG2* silencing upregulates the inflammation pathway. **A** Primary astrocytes were isolated and purified, transfected with siRNA, and subjected to RNA-seq after 8 h of OGD and lactate stimulation conditions (Created with www.BioRender.com). **B** qRT–PCR analysis of *NDRG2* mRNA after siRNA transfection. GAPDH was used as an endogenous control. Statistical significance was assessed using one‐way ANOVA. Data are expressed as means ± SD; *n* = 3, **P < 0.01 vs. siNC. **C** Western blot for NDRG2 protein expression after siRNA transfection. Statistical significance was assessed using one‐way ANOVA. Data are expressed as means ± SD; *n* = 3, **P < 0.01 vs. siNC. **D** Principal component analysis showing the overall transcriptomic similarity of the two groups. **E** Differential expression analysis reveals that *NDRG2* silencing induces transcriptional changes in astrocytes under OGD and high lactate conditions. Volcano plots show differentially expressed genes (DEGs; fold change > 1.5 or ≤ − 1.5, false-discovery-rate-adjusted *P*-value < 0.05) in astrocytes. **F** The heatmap of the top 50 downregulated (blue) and upregulated genes (red) significantly affected by *NDRG2* silencing in astrocytes. **G** GO analysis showing the top five terms significantly affected by *NDRG2* silencing, from the most downregulated (blue) to the most upregulated (red). **H** PPI networks among the upregulated DEGs and hub genes as computed by Cytoscape. Betweenness scores were used to screen hub genes. **I** qRT–PCR analysis verified the expression of representative genes discovered by RNA-seq analysis after siRNA transfection. GAPDH was used as an endogenous control. Paired *t*-tests were used for statistical comparisons. Data are expressed as means ± SD. *n* = 3, *P < 0.05 and **P < 0.01 vs. siNC

To study the *NDRG2* function in astrocytes, we comprehensively profiled the transcriptomic features of *NDRG2* silencing in astrocytes after 8 h of OGD conditions as well as after exogenous lactate treatment by performing RNA-seq (Fig. 3A). qRT–PCR and western blot results confirmed knockdown efficiency. siNDRG2-003 (small interfering nucleoprotein RNA) was chosen for subsequent experiments (Fig. 3B, C). Principal component analysis of the RNA-seq data showed that the transcriptome samples of siNC and si*NDRG2* clustered in two components, indicating a relatively small within-group variability for their respective transcriptomes (Fig. 3D).

Next, differential expression analysis was performed to determine the transcriptomic changes induced by *NDRG2* deletion in astrocytes. We identified 835 differentially expressed genes (defined as genes with fold changes of > 1.5 or ≤ − 1.5 and *P* < 0.05) in *NDRG2*-silenced astrocytes as compared with astrocytes in the control group (Fig. 3E). There were more upregulated than downregulated genes in the *NDRG2*-silenced astrocytes, suggesting that *NDRG2* deletion had an overall activating effect on transcription and may thus trigger the respective functions of these astrocytes.

The heat map of the top 50 downregulated and upregulated genes promoted by *NDRG2* silencing is presented in Fig. 3F. Several upregulated genes are associated with the regulation of immune system processes and inflammation (Additional file 2: Table S1). Gene ontology (GO) analysis revealed that downregulated gene-enriched GO terms were related to metabolism, and upregulated enriched GO terms were mostly associated with immune response (Fig. 3G). Moreover, 10 hub genes with a relatively high degree of connectivity were discovered by screening the protein–protein interaction (PPI) network of upregulated genes. All the above-mentioned hub genes and their biological functions are listed in Additional file 3: Table S2. Moreover, representative genes associated with inflammatory processes were validated by qRT–PCR, thus confirming the gene profiling results (Fig. 3H, I). Taken together, these results indicate that *NDRG2* silencing may have a meaningful impact on neuroinflammatory reactions in the progression of ischemic stroke.

### *NDRG2* knockdown upregulated inflammatory pathway activation

**Fig. 4:**
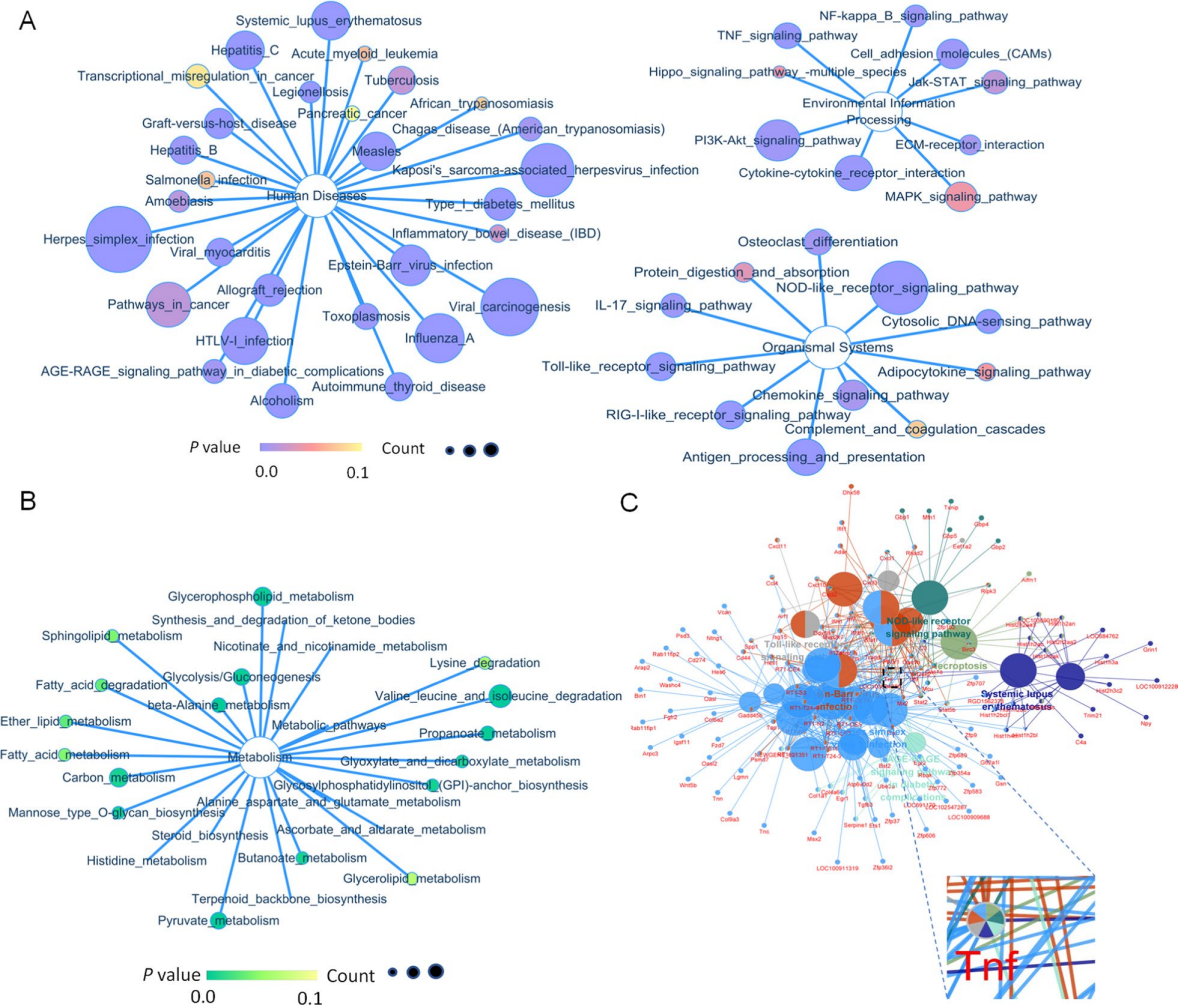
*NDRG2* knockdown upregulates inflammatory pathways in OGD. **A** KEGG analysis of the upregulated DEGs induced by *NDRG2* silencing in astrocytes predicts inflammation-associated canonical pathways. **B** KEGG analysis of the corresponding downregulated DEGs predicts inhibition of metabolism-associated canonical pathways. **C** The Cytoscape plugin ClueGO was used for KEGG pathway enrichment analysis and for determining clusters of signaling pathways. Area refers to the frequency of terms in the cluster. The terms are grouped into seven clusters. ClueGO was used to assess gene ontology and screen the central nodes

To explore the astrocyte functional changes induced by the differentially expressed genes (DEGs) more comprehensively, we first performed a signaling pathway classification analysis. The results showed that the main classifications of the upregulated pathways were human disease (immune disease and infectious disease), environmental information processing (signal transduction, signaling molecules, and interaction), and organismal systems (immune system, development, and regeneration) (Fig. 4A). The main classifications of the downregulated pathways were metabolism (amino acid metabolism, carbohydrate metabolism, lipid metabolism, glycan biosynthesis and metabolism, cofactors, and overall metabolism) (Fig. 4B). Additional signaling-pathway enrichment analyses demonstrated the enrichment of upregulated genes in seven clusters. *TNFα*, one of the hub genes mentioned above, appeared within the top enriched genes in all seven clusters, suggesting that *TNFα* might be essential for the extensive changes induced by *NDRG2* knockdown. In conclusion, the above results suggest that *NDRG2* knockdown upregulates inflammation-associated pathway activation and confirm that *TNFα* is a core downstream target gene regulated by *NDRG2* (Fig. 4C).

### *NDRG2* mediated the regulatory effects of lactate on TNFα expression

**Fig. 5:**
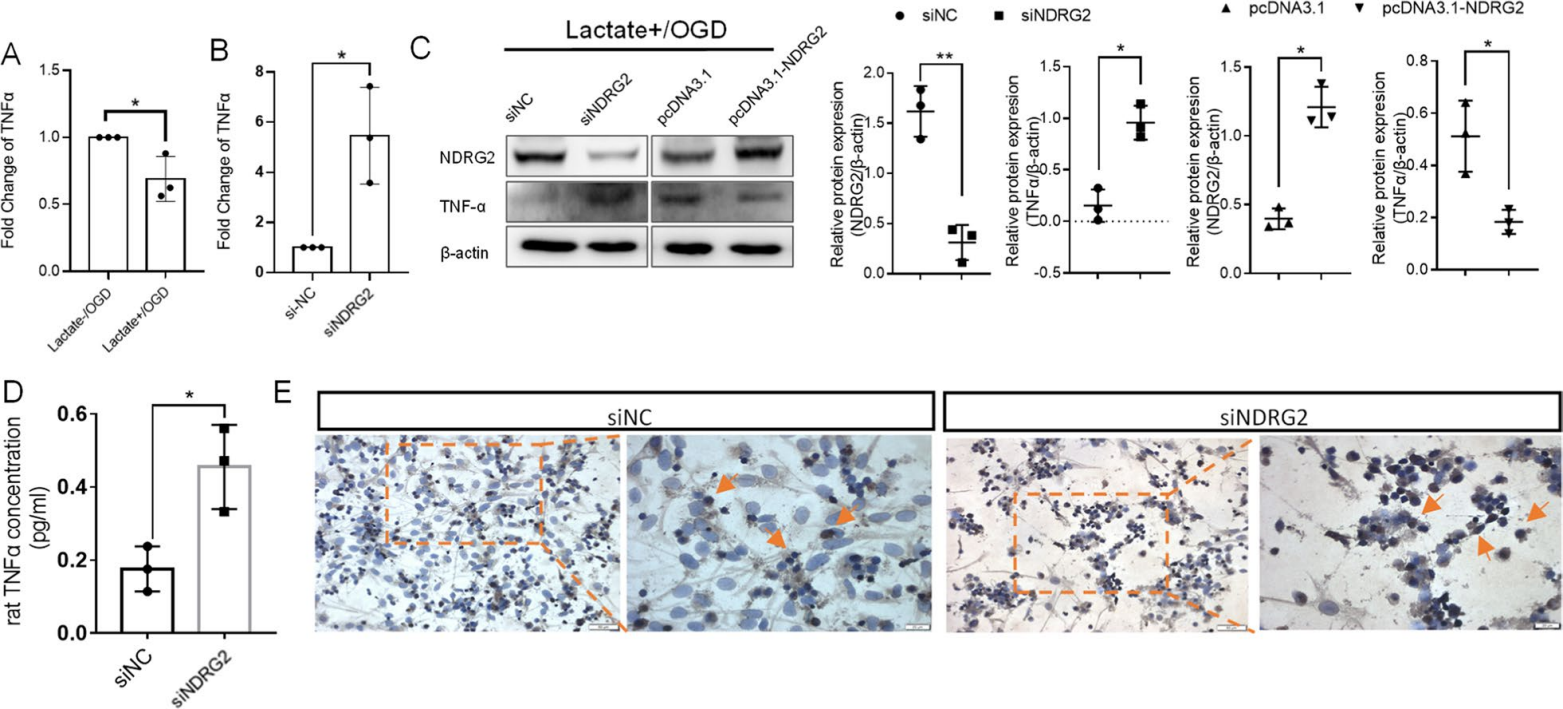
Lactate regulates TNFα expression in astrocytes through NDRG2 and affects the survival of nearby cells. **A** qRT–PCR analysis of *TNFα* mRNAs after lactate stimulation under OGD conditions. GAPDH was used as an endogenous control. Paired *t*-tests were used for statistical comparisons. Data are expressed as means ± SD. *n* = 3, *P < 0.05 vs. Lactate-/OGD. **B** qRT–PCR analysis of *TNFα* mRNAs after si*NDRG2* transfection under Lactate + /OGD conditions. GAPDH was used as endogenous control. Paired *t*-tests were used for statistical comparisons. Data are expressed as means ± SD. *n* = 3, *P < 0.05 vs. siNC. **C** Western blots for *NDRG2* and TNFα protein expression under Lactate + /OGD conditions. pcDNA3.1: control group, pcDNA3.1-*NDRG2*: *NDRG2* overexpression group. Paired t-tests were used for statistical comparisons. Data are expressed as means ± SD. *n* = 3, *P < 0.05 and **P < 0.01 vs. siNC in siRNA transfection group and *P < 0.05 vs. pcDNA3.1 in *NDRG2* overexpression group. **D** Extracellular TNFα content under Lactate + /OGD conditions measured via ELISA. Paired *t*-tests were used for statistical comparisons. Data are expressed as means ± SD. *n* = 3, *P < 0.05 vs. siNC. **E** TUNEL staining for intergroup differences in apoptosis (yellow arrows: apoptotic cells). Scale bar: 50 μm or 20 μm

To confirm the effects of lactate on *TNFα* as mediated by *NDRG2* pathways, we evaluated the regulation of *TNFα* by lactate. qRT–PCR results showed that, under OGD conditions, lactate meaningfully inhibited *TNFα* mRNA levels (Fig. 5A). Moreover, in the presence of lactate, *NDRG2* knockdown led to an increase in *TNFα* mRNA levels under OGD conditions (Fig. 5B). Western blot confirmed that TNFα protein levels increased after *NDRG2* knockdown and decreased after *NDRG2* overexpression mediated by plasmid transfection (Fig. 5C). To confirm that the levels of secreted TNFα protein were also affected by *NDRG2*, we measured the secreted TNFα levels in culture supernatants via an ELISA under the same conditions. The results showed that *NDRG2*-knockdown astrocytes secreted higher levels of TNFα (Fig. 5D). To determine whether this pro-inflammatory condition influences the survival of adjacent neurons, we cultured primary neurons with an astrocyte-conditioned medium (i.e., the culture supernatants mentioned above). A TUNEL assay detected a higher number of TUNEL-positive neurons after culturing with a conditioned medium from *NDRG2*-knockdown astrocytes (Fig. 5E). Moreover, according to our gene set enrichment analysis, apoptosis-associated genes tended to be enriched in *NDRG2*-silenced astrocytes under OGD and lactate treatment conditions (Additional file 1: Fig. S2A). A subsequent annexin V-FITC/PI (fluorescein isothiocyanate-annexin V/propidium iodide) assay showed that both early and late apoptotic cells increased after *NDRG2* knockdown. However, this increase was inhibited by the addition of a TNFα-neutralizing antibody (Additional file 1: Fig. S2B). Therefore, our results indicate that *NDRG2* knockdown promotes astrocyte apoptosis through TNFα and that *NDRG2* plays an important role in promoting cell survival.

**Fig. 6:**
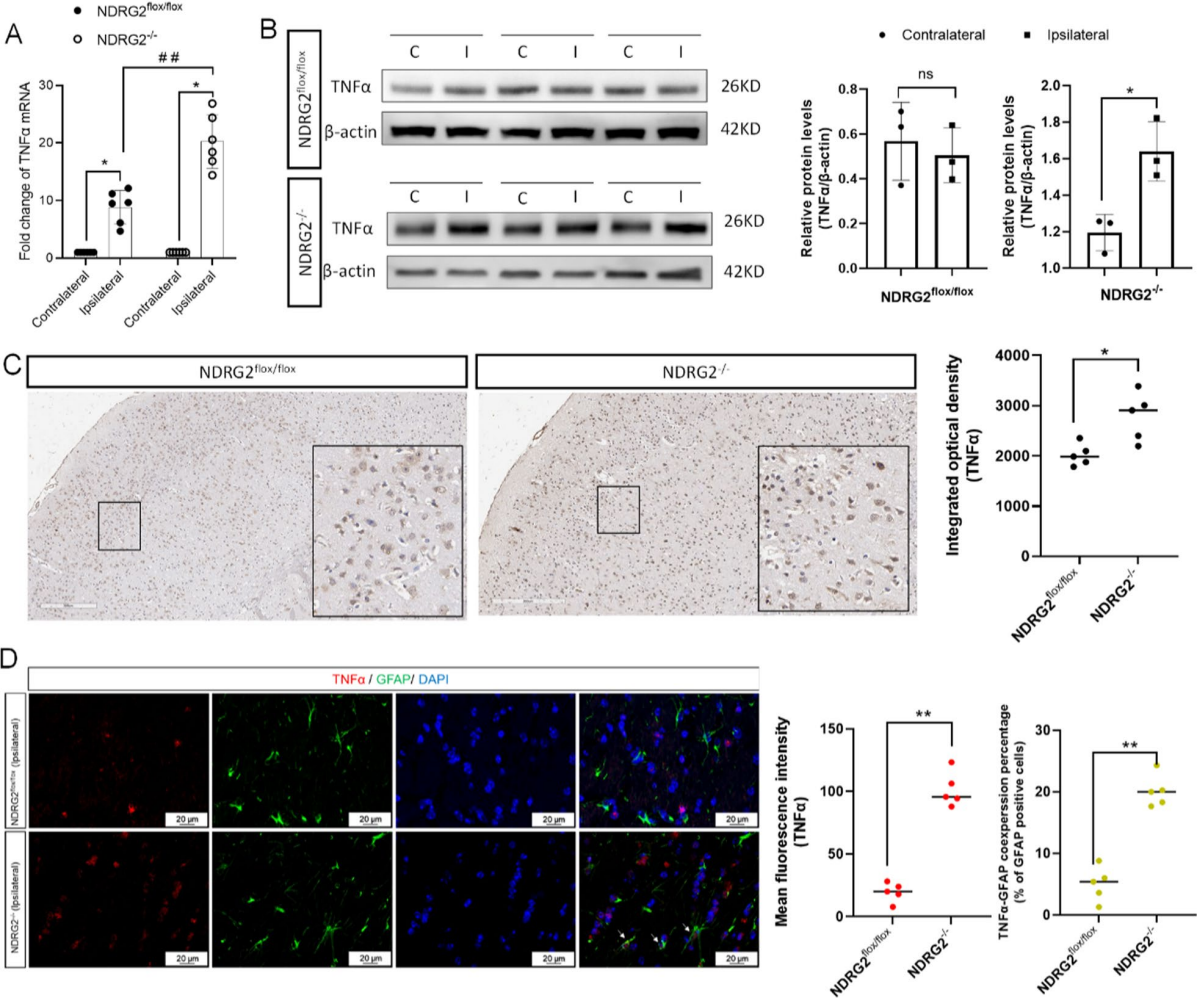
*NDRG2*^*−/−*^ mice have higher levels of TNFα in ischemic ipsilateral brain than *NDRG2*^flox/flox^ mice. **A** qRT–PCR analysis of *TNFα* mRNA in brain tissue. β-actin was used as an endogenous control. Paired *t*-tests were used for statistical comparisons. Data are expressed as means ± SD. n = 6, *P < 0.05 vs. Contralateral, ^##^*P* < 0.01 vs. Ipsilateral in *NDRG2*^flox/flox^. **B** Western blot for TNFα protein expression in brain tissue. C: Contralateral; I: Ipsilateral. Paired *t*-tests were used for statistical comparisons. Data are expressed as means ± SD. n = 3, ns = no significance, *P < 0.05 vs. Contralateral. **C** TNFα immunostaining in representative sections. Scale bar: 200 μm. Unpaired *t*-tests were used for statistical comparisons. Data are expressed as means ± SD. *n* = 5 random samples, *P < 0.05 vs. *NDRG2*^flox/flox^. **D** Representative brain sections obtained 8 h after MCAO surgery, double-stained for TNFα (red) and GFAP (green) (white arrows indicate double-stained cells). Scale bar: 20 μm. Unpaired *t*-tests were used for statistical comparisons. Data are presented as means ± SD. *n* = 5 random samples, **P < 0.01 vs. *NDRG2*^flox/flox^

To validate our findings more comprehensively, we conducted comparative evaluations of astrocyte-specific *NDRG2* knockout mice (GFAP-Cre *NDRG2*^flox/flox^ [*NDRG2*^−/−^]) and *NDRG2*^flox/flox^ littermate controls (Additional file 1: Fig. S3). Eight hours after MCAO, qRT–PCR and western blot results showed that tissue on the ipsilateral side of the *NDRG2*^−/−^ MCAO model expressed a higher level of TNFα than that in the *NDRG2*^flox/flox^ mice (Fig. 6A, B). This result is consistent with the TNFα immunohistochemical findings presented in Fig. 6C. The immunofluorescence double-staining results showed that astrocytes were not the main cells expressing TNFα after ischemia. However, compared with *NDRG2*^flox/flox^ mice, *NDRG2*^−/−^ MCAO model mice not only showed a significant increase in TNFα-positive cells at the injury site but also showed more GFAP-positive cells expressing TNFα (Fig. 6D). Taken together, the results confirm that lactate mediated the regulation of TNFα expression in astrocytes via *NDRG2*, thereby playing an important role in neuroprotection.

### *NDRG2* regulated TNFα expression through c-Jun phosphorylation

**Fig. 7:**
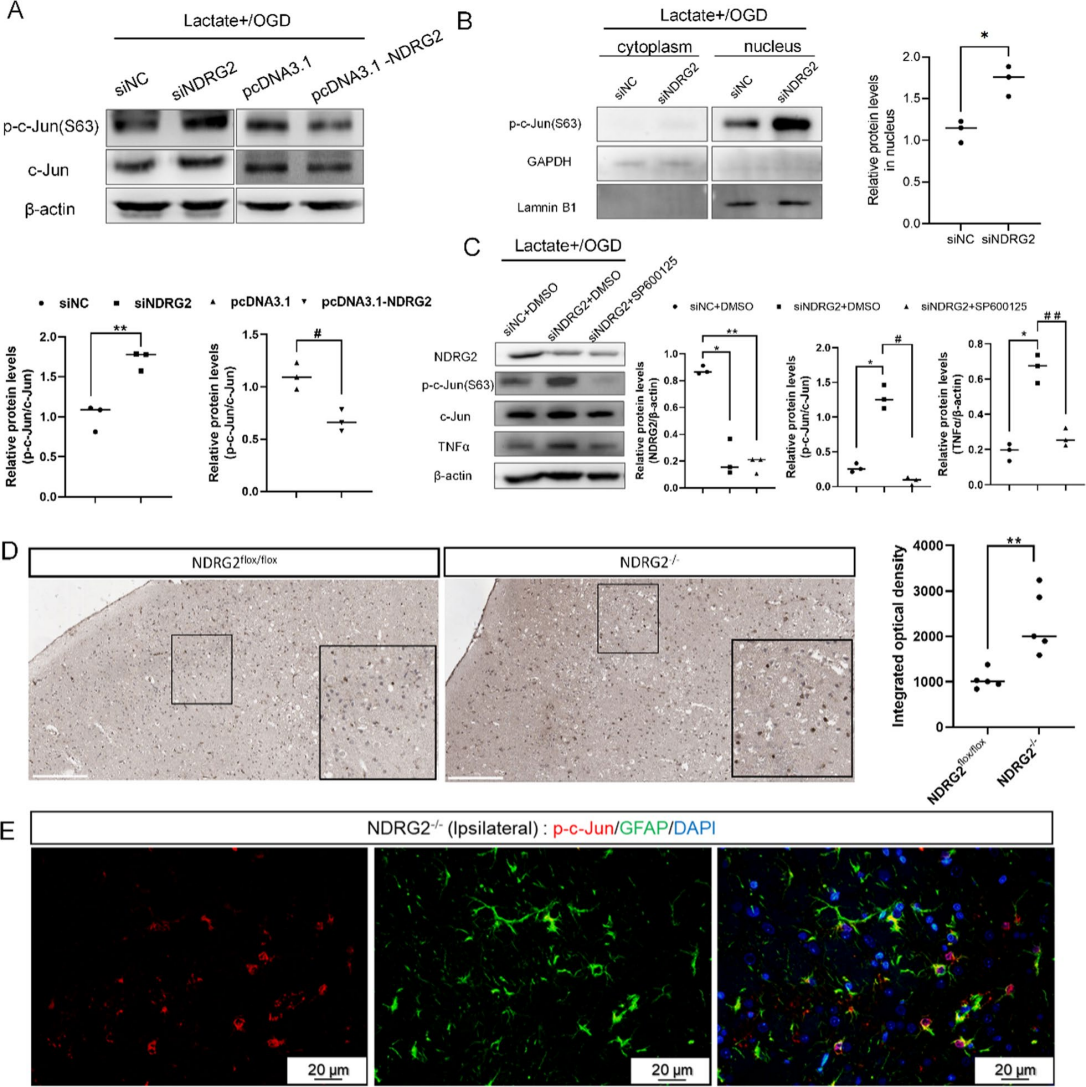
*NDRG2* regulates TNFα through p–c-Jun. **A** Western blot analysis of relative protein expression. pcDNA3.1: control group, pcDNA3.1-*NDRG2*: *NDRG2* overexpressed group. β-actin was used as a loading control. Paired t-tests were used for statistical comparisons. Data are expressed as means ± SD. *n* = 3, **P < 0.01 vs. siNC and ^#^*P* < 0.05 vs. pcDNA3.1. **B** Western blot for relative protein expression in cytoplasm and nucleus. GAPDH was used as a loading control for cytoplasmic proteins, and laminin B1 was used as a loading control for nuclear proteins. Paired t-tests were used for statistical comparisons. Data are expressed as means ± SD. *n* = 3, *P < 0.05 vs. siNC in nucleus. **C** Western blot analysis for relative protein expression. β-actin was used as a loading control. Statistical significance was assessed using one‐way ANOVA. Data are expressed as means ± SD; *n* = 3, *P < 0.05 and **P < 0.01 vs. siNC + DMSO, ^#^*P* < 0.05 and ^# #^*P* < 0.01 vs. siNDRG2 + DMSO. **D** p–c-Jun immunostaining in representative sections. Scale bar: 200 μm. Unpaired t-tests were used for statistical comparisons. Data are presented as means ± SD. *n* = 5 random samples, ** *P* < 0.01 vs. *NDRG2*^flox/flox^. **E** Representative brain sections obtained 8 h after MCAO surgery, double-stained for p–c-Jun (red) and GFAP (green). Scale bar: 20 μm

To evaluate the molecular mechanisms of *NDRG2* in regulating TNFα under OGD conditions more comprehensively, we evaluated the potential regulation of *NDRG2* with respect to c-Jun phosphorylation (a hallmark of c-Jun activation). C-Jun is a member of the AP-1 transcription factor family and has been implicated in the transcription of myriad inflammatory cytokines, including TNFα. We examined whether *NDRG2* depletion upregulated TNFα expression by inducing c-Jun phosphorylation. Western blot results showed that phosphorylated c-Jun increased after *NDRG2* depletion and was abrogated by *NDRG2* overexpression; the total c-Jun levels were consistent with these trends (Fig. 7A). Additional experiments showed that increased phosphorylated c-Jun was mainly located in the nucleus (Fig. 7B). In addition, we found that treatment with SP600125, a well-established JNK (c-Jun N-terminal kinase) inhibitor, reduced the levels of phosphorylated and total c-Jun (as expected) and likewise inhibited TNFα expression (Fig. 7C). Immunohistochemistry results showed that the tissue from the ipsilateral side of the *NDRG2*^*−/−*^ MCAO model expressed a higher level of phosphorylated c-Jun (p–c-Jun) in the nucleus than that of the *NDRG2*^flox/flox^ mice (Fig. 7D). In addition, double-staining immunofluorescence results showed that almost all the p–c-Jun was expressed by GFAP-positive astrocytes (Fig. 7E). In summary, these results indicate that *NDRG2* expression closely regulates TNFα levels under OGD conditions by regulating c-Jun transcription and phosphorylation.

## Discussion

In this study, we evaluated *NDRG2*-mediated lactate signaling and its respective neuroprotective roles in astrocytes under OGD conditions. We found that high levels of lactate increased the expression levels of intracellular *NDRG2*. Additionally, we found that *NDRG2* deficiency triggered the dysregulation of inflammatory and immunomodulatory activity and led to elevated levels of TNFα, thus leading to additional destruction of the local microenvironment.

Elucidating the mechanisms during early-stage of ischemic stroke injury is important to develop new strategies capable of minimizing detrimental side effects and improving patient outcomes following stroke. Previous studies in humans indicate that the lactate signal within the infarct area is initially significantly elevated and then decreases sharply. Finally, lactate levels appeared to normalize at 2 weeks [21, 22]. Similarly, studies using rat MCAO models suggest that lactate levels are significantly higher than baseline within a few minutes after MCAO onset and return to normal at 48 h [23–25]. Therefore, we speculate that lactate may play an important role, mainly in the early stages of the onset of ischemic stroke, especially in the first few hours. However, the accumulation of lactate may take longer to significantly affect the associated phenotype of astrocytes. In combination with our previous findings from model of a rat with MCAO, we demonstrated that *NDRG2* protein levels in ipsilateral tissue of MCAO models show an increasing trend over time, but the difference detected by western blot compared with the contralateral side is statistically significant, at least 8 h later, during which it is accompanied by astrocyte activation [8]. The lactate content of the injured side is also significantly higher than that of the contralateral side at 8 h [8]. Therefore, we believe that 8 h is the key time point for the detection of related indicators in vivo and in vitro, and, in the subsequent experiments in this manuscript, were uniformly detected at 8 h.

In a high-lactate microenvironment, lactate either mediates signaling via its receptor expressed on the cell surface or is transported within the cells where it mediates signal transduction. GPR81 (also named hydroxycarboxylic acid receptor 1 [HCAR1]), an orphan G-protein-coupled receptor, is a sensor of extracellular lactate and modulates cell functions during oxygen-deficient conditions [26]. Moreover, it is also reported to suppress inflammation (even the production of TNFα and IL6) and is involved in the protection of the brain from the deleterious effects of ischemic stroke [27, 28]. qRT–PCR analysis revealed the presence of *GPR81* mRNA in astrocytes, although at low levels compared to neurons and other tissues such as fat [29]. Therefore, we cannot rule out that the effects of lactate may also involve the potential contribution it plays through GPR81 function. Further, our previous studies demonstrated that extracellular lactate might execute its role under OGD conditions by transport into astrocytes (mediated via MCT1) [8]. In our current study, we showed that the *NDRG2* mRNA levels were genetically independent of lactate. In addition*, NDRG2* protein levels were upregulated by lactate treatment and decreased when the lactate uptake was suppressed by siMCT1. Therefore, we speculate that intercellular lactate plays a major role in regulating *NDRG2* expression at the translational level or post-translational level.

To date, direct protein targets of intercellular lactate have rarely been reported. However, a previous study identified that *NDRG3*, another member of the *NDRG* family, acts as a lactate sensor whose ubiquitination and degradation are inhibited by direct lactate binding through hydrogen bonds, thus triggering downstream kinase signaling and providing the genetic basis for lactate-induced hypoxia responses [30]. Given the high degree of primary amino acid sequence and structural similarities among *NDRG* family members, we also studied the effects of lactate on the ubiquitination of *NDRG2* [31]. The present study demonstrates that lactate suppresses *NDRG2* ubiquitination, leading to the accumulation of intercellular *NDRG2*; this process mostly occurs through direct binding (which mainly comprised hydrogen bonds). These results indicate that the existence of *NDRG2*-mediated signaling is enhanced under OGD conditions along with lactate-induced astrogliosis. This occurs even in the energy-restricted state of cerebral ischemia since the NDRG2 protein, once stabilized by lactate binding, remains quite stable. However, recently, accumulating evidence has proven that, at high lactate levels, lactylation occurs on the lysine residues of cellular histone proteins (i.e., in macrophages and other cancer cells) [32–34]. This new type of post-translational protein modification plays a crucial biological function during physiological and pathological conditions. Hence, we cannot exclude the possibility that lactate also exerts this function through the NDRG2 lactylation. Additional experiments are needed to explore the lactylation of NDRG2 and its resulting functions.

NDRG2 is emerging as a critical and promising neuroprotection target against cerebral ischemic injury through different mechanisms. A series of studies have suggested that NDRG2 played important roles in maintaining the integrity of the blood–brain barrier (BBB), alleviating brain edema, and inhibiting glutamate excitotoxicity after ischemic stroke [15, 16, 35]. Our current study complementally confirms the importance of *NDRG2* in attenuating astrocytic neuroinflammation. Our RNA-seq demonstrated that *NDRG2* silencing leads to the upregulation of a large number of inflammatory factors and chemokines, as well as the upregulation of inflammation-associated signaling pathways. C3 and RT1-S3 (H2-T23 [histocompatibility 2, T legion locus 23]), which are markers of the A1 phenotype, are each associated with hub genes in the PPI network of upregulated genes after NDRG2 silencing [36], in addition to a plethora of pro-inflammatory factors and chemokines, such as CXCL10, CXCl11, IL12a, and TNFα. These effects contribute to a neurotoxic microenvironment. Liddelow et al. previously treated mouse models with systemic injection of lipopolysaccharide (LPS) or induced MCAO to cause ischemia. These two treatments resulted in two different astrocytic phenotypes (termed A1 and A2), each associated with a specific subset of upregulated transcripts [37]. Given that Sprague–Dawley rats and mice are closely related, we analyzed whether the *NDRG2* knockdown in rats affected the expression of the A1/A2-specific transcripts previously defined in mice. We found a relatively higher ratio of A1-specific transcripts (17.1%) than A2-specific transcripts (10%) in the upregulated genes after *NDRG2* silencing (Additional file 1: Fig. S4). Taken together, our results suggest that *NDRG2* silencing leads astrocytes to an A1-like polarization propensity, if not to a complete A1 subtype. Therefore, we conclude that elevated lactate levels under ischemic conditions prevent A1 subtype astrocyte formation and modulate a beneficial microenvironment to promote cell survival by maintaining *NDRG2* stability.

TNFα is a well-known pro-inflammatory cytokine that plays an important role in inflammatory disorders. Through enrichment analysis of signaling pathways, we identified a key “common signaling node”, namely TNFα, that was associated with all seven classifications of signaling pathways, identifying TNFα as a bridging gene. Therefore, in the current study, we focused on the regulation of TNFα as mediated by NDRG2. TNFα exerts its effects through either pro-death or pro-survival/inflammation-associated pathways [38, 39]. Moreover, TNFα triggers these effects through two structurally related but functionally distinct receptors: the type 1 receptor (TNF-R1) (p55) and the type 2 receptor (TNF-R2) (p75). TNF-R1 contains a cytoplasmic death domain that recruits downstream signaling proteins through the adaptor protein TRADD and initiates subsequent processes including inflammatory, apoptotic, and degenerative cascade effects [40, 41]. Our qRT–PCR assay showed that *NDRG2* silencing increased mRNA levels of TNFα, TNFR1 (TNFRSF1a), and TRADD. In addition, *NDRG2* knockdown promotes astrocyte apoptosis through TNFα (Additional file 1: Fig. S2). Our study thus provides evidence that *NDRG2* deficiency is not favorable for cell survival as it induces TNFα expression and secretion. Furthermore, TNF-R1 is distributed throughout the brain and is expressed in many types of cells, including microglia and neurons [42, 43]. Astrocyte-derived TNFα has been reported to critically modulate microglial activation and M1-like polarization [44, 45]. In addition, we confirmed by TUNEL assay that the cell supernatant of *NDRG2*-silenced astrocytes could promote neuronal apoptosis. These findings suggest that *NDRG2* plays an important role in promoting cell survival and inhibiting inflammation following ischemia, thus contributing to a favorable microenvironment that is beneficial for neural restoration.

However, this study had several limitations. First, although we demonstrated the possibility that lactate inhibits NDRG2 ubiquitination through direct interaction and predicted that Lys176 is the key amino acid residue mediating this interaction, our experimental design did not lend itself to providing direct evidence supporting this putative mechanistic pathway. An in vitro binding experiment evaluating ^14^C-labeled L-lactate combined with *NDRG2* would help clarify these findings. Second, we showed that *NDRG2* knockdown upregulated inflammation-associated pathway activation, with TNFα as the core component. However, this finding does not signify that TNFα plays the most important role in mediating the mechanistic effects of *NDRG2.* Additional systematic experiments will be required to determine the exact functions of *NDRG2* and TNFα in regulating astrocytic phenotypes and in the outcome of cerebral ischemia. Third, behavioral and histological data for *NDRG2* knockout mice in MCAO models are lacking. Based on our experience, differences between animal models due to responses to *NDRG2* might be offset by the effects of serious injury occurring after 8 h of MCAO. Thus, future studies are needed to acquire more thorough information on a range of animal models to evaluate the role of lactate and NDRG2 following acute insult in MCAO (i.e., 1 h or 2 h blocking) and reperfusion conditions.

## Conclusions

**Fig. 8:**
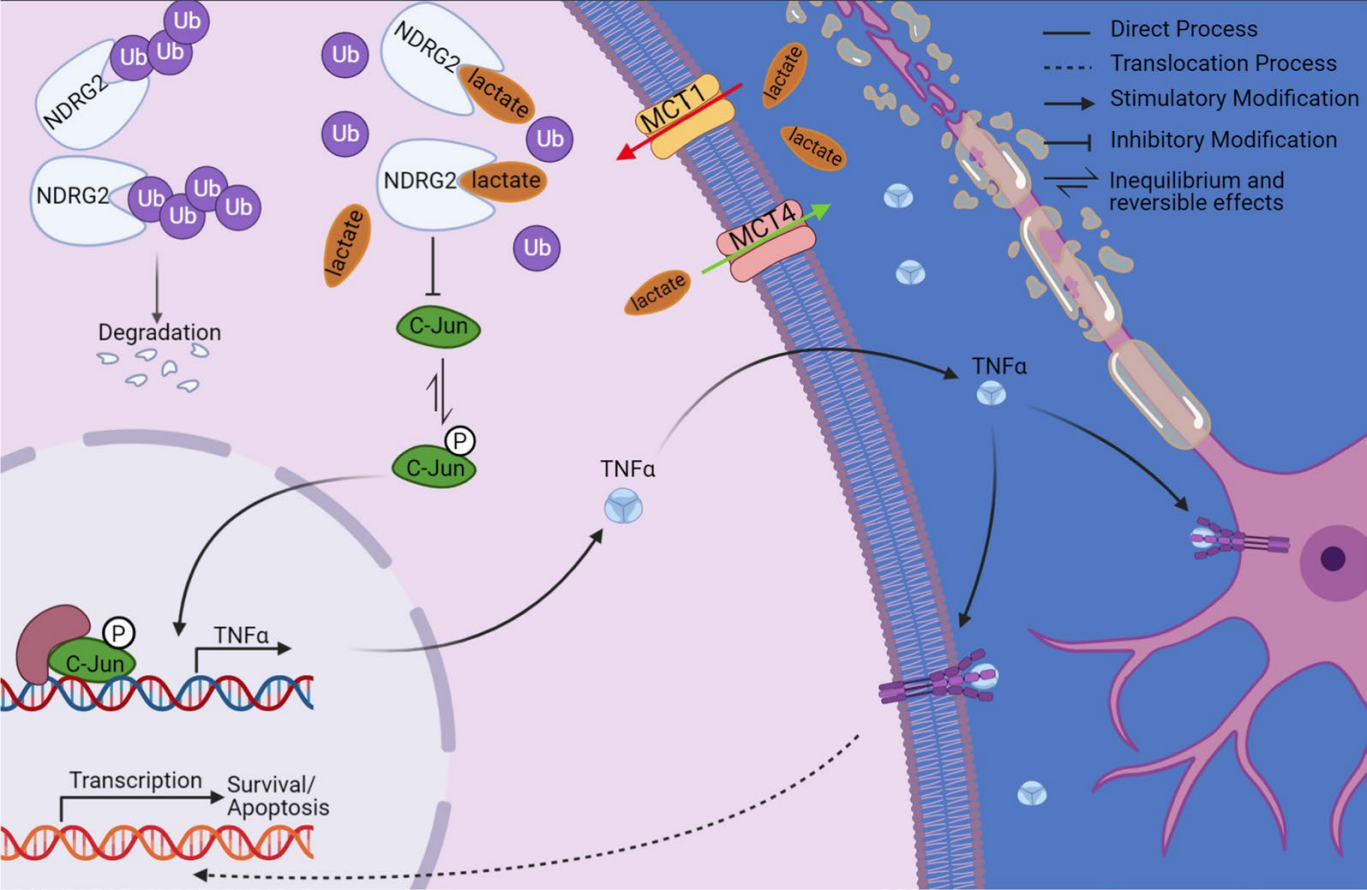
Mechanism of neuroinflammation regulation by lactate via *NDRG2* (N-Myc downstream-regulated gene 2) and TNFα. Phosphorylated c-Jun promotes the expression of TNFα, which exacerbates neuroinflammation and damages neighboring cells. However, lactate directly binds with *NDRG2* and inhibits its ubiquitination and degradation. *NDRG2* inhibits c-Jun phosphorylation and thus subsequently inhibits TNFα production. This finding suggests a valuable approach for modulating neuroinflammation during the early stages of ischemia (Created with www.BioRender.com)

Overall, we found that lactate-mediated effects on reactive astrocytes displayed a neuroprotective role during the early stages of ischemia as well as under OGD conditions. Our findings emphasize the beneficial functions of lactate and its critical anti-inflammatory role as well as in promoting cell survival; these roles are mediated by maintaining astrocytic *NDRG2* stability (Fig. 8). In this context, we provide critical information on a newly reported mechanism, demonstrating that lactate acts as a signaling molecule in ischemia. We conclude that the lactate/*NDRG2*/TNFα signaling axis may provide an extended mechanistic clue and a valuable approach for modulating neuroinflammation during the early stages of ischemia. Our findings thus inform future research directions and, if confirmed, will directly inform medical guidelines.

## Methods

### Experimental model and subject details

The adult male Sprague–Dawley rats (aged 7–10 weeks and weighing 200–240 g) used in this study were supplied by the Experimental Animal Center of the Jilin Yisi Company (Chang Chun, China). *NDRG2*^flox/flox^ and *NDRG2*^−/−^ astrocyte conditional knockout (CKO) mice with a C57BL/6 background were generated by gene targeting in embryonic stem cells (Cyagen Bioscience Inc.; Guangzhou, China). *NDRG2*^flox/flox^ mice were crossed with GFAP-Cre mice to generate *NDRG2*^−/−^ CKO mice. The resulting *NDRG2*^flox/flox^ mice and *NDRG2*^−/−^ mice were viable, fertile, and showed no obvious phenotypes. The mice were used in the experiments at 6–8 weeks of age. All animals were housed in a specific pathogen-free, temperature-controlled facility with a regular 12-h light–dark cycle and ad libitum access to food and water.

### Method details

#### MCAO

Focal cerebral ischemia was induced by MCAO via the thread embolism method for 8 h in male rats and mice. MCAO was performed as previously described [8]. Briefly, rats were anesthetized intraperitoneally with 3% pentobarbital sodium (1–2 mL/kg), and mice were anesthetized with 1% pentobarbital sodium (10 mL/kg) until no pinch-paw reflex was observed. Body temperature was maintained at 37 °C using an electric blanket. The experiments were performed in a blinded manner. A vertical midline cervical incision was performed, and the right common carotid artery (CCA) was exposed and ligated at its proximal end. All branches of the external carotid artery (ECA) were isolated, coagulated, and transected. The internal carotid artery (ICA) was isolated, and the pterygopalatine artery (PPA) was ligated close to its origin. The CCA was ligated with a slipknot in the middle of the origin of the PPA and ECA to block blood flow temporarily. A slot was cut in the CCA above the ligation near the proximal end, and a thread embolism (Cinontech, Beijing, China) was inserted along the CCA. A slipknot was made above the cut to prevent leakage. The slipknot was opened between the PPA and ECA, and the thread embolism was advanced through the ICA until resistance was felt, occluding at the origin of the middle cerebral artery. The slipknot was tightened to fix the thread embolism. The ipsilateral side was analyzed, and the contralateral side of the same rat or mouse was used as the control.

#### Cell isolation and cell culture

Primary astrocyte isolation was performed as described previously [8]. Briefly, the cortices of newborn Sprague–Dawley rats 1–3 days old were dissociated in DMEM/F12 (1:1) medium (Hyclone Laboratories, Inc., Logan, UT, USA; SH30023.01) and digested in 0.05% trypsin/EDTA (ethylenediaminetetraacetic acid; Gibco, Waltham, MA, USA; 25200056) with 1% DNAse (Worthington Industries Medical Center, Inc., Columbus, OH, USA; IDLS006331) at 37 °C for 25 min to yield a single-cell suspension. Cells were collected and resuspended in DMEM/F12 (Dulbecco’s modified Eagle’s medium/nutrient mixture F-12; 1:1) medium containing 10% fetal bovine serum (FBS; Sigma, Australia, F8318) and plated in culture flasks. After 5 days, the other cell types (oligodendrocytes and microglia) growing on top of the astrocytes were removed by shaking the flasks in an orbital shaker for 16 h. Cells were passaged once they reached over 90% confluence using 0.25% trypsin/EDTA. Cells at passage 1 were used for the experiments.

#### OGD

We used an in vitro OGD model to mimic ischemic conditions. Hypoxic (< 0.1% oxygen) culture conditions were achieved using the Anaero Pack system in a 2.5 L jar (MGC, Tokyo, Japan); this system works as an oxygen absorber and CO_2_ generator. The composition of the gas mixture reached approximately 78% N_2_ and 22% CO_2_ within 1 h, as measured by the color change of the oxygen indicator, and the pressure inside the jar was equal to that outside the jar. The ‘lactate + ’ culture medium contained DMEM without glucose (Gibco, 11966025), as well as 30 mM L-lactate sodium (Sigma, St. Louis, MO, USA; 71718), 1 mmol/L sodium pyruvate (Sigma; P5280), 15 mM HEPES (N-2-hydroxyethylpiperazine-N′-2-ethanesulfonic acid, Sigma, H4034), and FBS (fetal bovine serum; 1%). The pH was set to 6.87 ± 0.02.

#### Cell transfection

Astrocytes were seeded into six-well plates at a density of 5 × 10^5^ cells per well 24 h prior to transfection. For siRNA-mediated gene knockdown, three different siRNAs targeting *NDRG2* (RiboBio) were delivered using Lipofectamine RNAiMAX (Thermo Fisher, Waltham, MA, USA; 13778075) following the manufacturer’s protocol. Since the silencing efficiencies of all three siRNAs were found to be similar, one of the siRNAs was randomly selected for subsequent experiments.

NDRG2 was overexpressed by the transfection of cells with pcDNA3.1-NDRG2 plasmid DNA, which was accompanied by pCDNA3.1 as the control group. pcDNA3.1 and pcDNA3.1-NDRG2 were synthesized by PPL (Public Protein/Plasmid Library, China). The cell transfection was delivered using the X-tremeGENE HP DNA Transfection Reagent (Roche, Basel, Switzerland; 06366244001) following the manufacturer’s protocol. Plasmids with site-directed mutations were purchased from PPL (Public Protein/Plasmid Library, China). Different forms of *NDRG2*-overexpressing plasmids were constructed using the pcDNA3.1 vector containing *Rattus norvegicus NDRG2* with a C-terminal 3 × Flag-tag (DYKDDDDK-tag). A Lys176 single-mutation plasmid and an Arg240 single-mutation plasmid were constructed by changing the relevant codon to a GCA codon encoding alanine. For the *NDRG2* overexpression vector, three forms of *NDRG2*-overexpressing plasmids (wild-type [WT], site-directed mutagenesis of Lysine 176 to alanine [K176A], double site-directed mutagenesis of Lysine 176 to alanine and Arginine 240 to alanine [K176A + R240A]) were delivered using the X-tremeGENE HP DNA Transfection Reagent.

#### qRT–PCR

Total RNA was isolated from astrocytes or tissues using TRIzol reagent (Invitrogen, Waltham, MA, USA). For tissues, the animals were killed at pre-specified time points, and the brains were rapidly removed and coronally sliced into 2-mm sections in cold phosphate-buffered saline (PBS). The ipsilateral side was analyzed, and the contralateral side of the same rat or mouse was used as the control. Both sides were quickly frozen with liquid nitrogen until they cracked, after which they were ground in TRIzol. Total RNA was extracted using the miRNeasy Mini Kit (Qiagen, Hilden, Germany), and cDNA was synthesized using TransScript One-Step gDNA Removal and cDNA Synthesis SuperMix (Transgene, Strasbourg, France) according to the manufacturer’s instructions. Briefly, the reaction was performed in a final volume of 20 μL with 500 ng of total RNA, 10 μL of 2 × TS reaction mix, 1 μL of oligo_(dT)_^15^, 1 μL of gDNA remover, and 1 μL of RT/RI enzyme mix, followed by incubation at 42 °C for 30 min and at 85 °C for 5 s. RT-qPCR was conducted using TransStart Green qPCR SuperMix (Transgen, Beijing, China) in an ABI7300 qRT–PCR machine. The primers used are listed in Additional file 4: Table S3. Gene expression was normalized to that of the internal control (β-actin) using the 2^−ΔΔCt^ method.

#### Western blot analysis

Total protein content was extracted from the cultured cells or tissues, and protein concentrations were determined using the Pierce BCA assay kit (Beyotime, Haimen, China; P0012S). Antibodies against GFAP (1:10,000; Abcam, Cambridge, UK; ab7260), NDRG2 (1:5000; Sigma-Aldrich; HPA002896), p-STAT3 (1:2000; CST, Naples, FL, USA; 9145P), STAT3 (1:2000; CST, 4904 T), p-Akt (1:2000; CST, 4060S), Akt (1:2000; CST, 9272S), c-Jun (phospho S63) (1:1000, CST, 91952 T), c-Jun (1:1000, CST, 9165 T), TNFα (1:500, Abcam, ab6671), HIF1α (1:1000, Sigma, SAB5200017), GAPDH (1:2000; CMC-TAG, AT0002) and β-actin (1:2000; CMC-TAG, AT0001) were used for western blot analysis. The band intensities of the target proteins were quantified by densitometry using ImageJ software (National Institutes of Health, Bethesda, MD, USA). GAPDH and β-Actin were used as the loading control. The results were derived from three independent experiments.

#### Nuclear and cytoplasmic protein extraction

Nuclear and cytoplasmic proteins were extracted using the Nuclear and Cytoplasmic Protein Extraction Kit (Yeasen, Shanghai, China) according to the manufacturer’s instructions. In brief, cells were washed and harvested by scraping in cold PBS. Cell pellets were obtained after centrifugation. 200 µL of reagent A containing PMSF was added per 20 µL cell precipitation. The sample was vortexed vigorously (highest setting on vortex) for 5 s, then incubated for 15 min in an ice bath. Subsequently, 10 µL of cytosolic protein extraction reagent B was added to the samples and the mixture was vortexed vigorously for 5 s, then incubated for 1 min in an ice bath. Samples were centrifuged at 12,000–16,000*g* for 5 min at 4 °C. The supernatant was the extracted cytoplasmic protein. 50µL of reagent C containing PMSF was added to the precipitate, then the mixture was incubated for 30 min in an ice bath. Samples were centrifuged at 12,000–16,000*g* for 10 min at 4 °C. The supernatant was the extracted nuclear protein, which was immediately transferred into a pre-cooled tube.

#### Histological and immunofluorescence staining

Rats and mice were transcardially perfused with 10% neutral formalin buffer, and the brains were dissected and post-fixed in 10% neutral formalin buffer for an additional 48 h. The fixed samples were embedded in paraffin and sectioned into 5-μm-thick sections. For immunohistochemistry, the sections were deparaffinized, rehydrated, washed with PBS several times, and treated with an UltraSensitive™ SP (mouse/rabbit) immunohistochemistry (IHC) kit (Maixin Biotechnology, Fuzhou, China; KIT-9710). The sections were incubated with rabbit anti-GFAP antibody (1:1000; Abcam, ab7260), rabbit anti-NDRG2 antibody (1:5000; Sigma-Aldrich, HPA002896), rabbit anti-TNFα antibody (1:200, Novus, St. Louis, MO, USA; NBP1-19532), and rabbit anti-c-Jun (phospho S63) antibody (1:100; Abcam, ab32385), and the nuclei were counterstained with hematoxylin. The stained sections were examined using the Aperio Digital Pathology Slide Scanner (Leica, Wetzlar, Germany) and an ImageScope imaging system (Leica). The integrated optical densities were semi-quantified using Image-pro Plus software (version 6.0; Media Cybernetics, Rockville, MD, USA). The following antibodies were used for fixed cells: rabbit anti-c-Jun (phospho S63) antibody (1:100; Abcam, ab32385), chicken anti-GFAP antibody (1:1000; Abcam, ab4674), chicken anti-MAP2 antibody (1:1000; Abcam, ab5392), Alexa Fluor 594 goat anti-rabbit immunoglobulin G (IgG) (1:200; Jackson Healthcare, Alpharetta, GA, USA; 140418), and Alexa Fluor 488 goat anti-chicken IgG (1:500; Jackson, 103545155). Nuclei were counterstained with Hoechst 33342 (1:1000; Beyotime, C1025). Sections were examined using a fluorescent microscope (Olympus, Tokyo, Japan) and the CellSens Entry imaging system (Olympus).

#### Molecular structure modeling and molecular docking simulation

A 3D model of NDRG2 was constructed using I-TASSER software (Iterative Threading ASSEmbly Refinement) [46], resulting in a high-value model (C‐score = 1.37, estimated TM‐score [template modeling score] = 0.90 ± 0.06, estimated RMSD [root-mean-square deviation] = 3.3 ± 2.3 Å). The lactate structure was downloaded from the RCSB Protein Data Bank (http://www.rcsb.org/). Based on binding-change mutations at Gly146, Asp172, Lys176, Asp180, and Arg240, lactate was docked into a 15-Å binding pocket formed by the five amino acids of NDRG2 (Additional file 1: Fig. S5). A comparative molecular docking analysis between lactate and NDRG2 was carried out using AutoDock Vina open-source software (Trott et al. 2010).

#### Ubiquitination assays

To conduct the in vivo ubiquitination assay, ‘lactate-’ or ‘lactate + ’ culture medium was generated by excluding or adding (respectively) the proteasome inhibitor MG132 (10 μM, Selleck, Berlin, Germany; S2619) from the culture during 8 h of OGD conditions prior to harvesting. Cell lysates were precleared by adding 50 μL of protein A/G immune magnetic beads (Bimake, Houston, TX, USA; B23201) and were immunoprecipitated with anti-NDRG2 antibody. Polyubiquitinated forms of NDRG2 were detected by western blot with an anti-ubiquitin antibody (1:1000, Cell Signaling Technology, A100). Astrocytes were transfected with expression vectors (i.e., different forms of Flag-tagged *NDRG2* overexpressing vector [WT, K176A, K176A + R240A]); 72 h later, the medium was changed to a ‘lactate + ’ culture medium by administering MG132 for 8 h under OGD conditions prior to harvesting. Cell lysates were precleared by adding 20 μL of anti-Flag-conjugated magnetic beads (Bimake, B26101) and were subsequently immunoprecipitated with anti-NDRG2 antibody. Proteins immunoprecipitated with anti-Flag magnetic beads were detected by western blot using an anti-ubiquitin antibody.

#### RNA-seq

Total RNA isolation was performed using the NEBNext Poly (A) mRNA Magnetic Isolation Module (New England Biolabs, Ipswich, MA, USA). The libraries were prepared using a KAPA Stranded RNA-Seq Library Prep Kit (Illumina, San Diego, CA, USA), and were sequenced to a mean depth of (19.7 ± 1.7) million reads using an Illumina HiSeq 4000 sequencer (Illumina) for 150 cycles. RNA-seq data were analyzed using R software (version 3.5.3; The R Project for Statistical Computing, Vienna, Austria) ballgown package, and were expressed as fragments per kilobase of transcript per million mapped fragments (FPKM). Bioinformatics analyses were performed using R and Python (version 3.4.10; Fredericksburg, VA, USA). DEGs in the two groups were identified using FPKMs and *P*-values (fold change > 1.5, *P*-value < 0.05). Hierarchical clustering was performed to extract distinguishable mRNA expression profiles from the samples. Gene ontology (GO) analysis was performed to investigate three functional domains, namely biological process (BP), cellular component (CC), and molecular function (MF). Pathway analysis was performed to functionally analyze and map the DEGs to KEGG pathways. *P*-values < 0.05 identified statistically significant GO terms and KEGG pathways correlated with various conditions.

#### ClueGO clustering analysis

The Cytoscape plugin ClueGO (https://www.cytoscape.org) was used for the functional enrichment analysis. Specifically, ClueGO was implemented to decipher functionally grouped gene ontology and pathway annotation networks. In this calculation process, a kappa coefficient was calculated to reflect the functional correlation between paths or terms based on gene overlap between pathways. Network specificity was scaled to the average.

In reporting the results, functionally similar entries are displayed in the same color. The threshold for enrichment significance was set to *P* < 0.05. Functionally grouped networks of enriched categories were generated for the candidate target genes. GO terms are represented as nodes, the lines between nodes reflect correlations between signaling pathways, and node size represents enrichment significance. The colors of the nodes reflect the enrichment classification of each node (i.e., which functional group it belongs to), and node pie charts represent the biological processes for these targets.

#### ELISA

To detect concentrations of secreted TNF, cell supernatants were assayed for TNFα proteins using ELISA after removing particulates via centrifugation. Eight hours after lactate treatment under OGD conditions, the culture supernatants were harvested and stored at − 70 °C. The concentrations of TNFα were measured using ELISA kits according to the manufacturer’s instructions (R&D Systems, Minneapolis, MN, USA; RTA00). The sample results were scaled to those of the siNC group.

### Quantification and statistical analysis

Results are expressed as means ± standard deviations. The sample sizes are shown in each figure. Statistical analyses were performed using GraphPad Prism software (version 7.0; San Diego, CA, USA). The parametric paired t-test was used on pairs of related samples and parametric unpaired t-test was used on pairs of independently sampled groups. Comparisons of more than two groups were conducted by one-way ANOVA with Tukey’s multiple comparison test. *P* values < 0.05 were considered statistically significant and are labeled with single asterisks (*P < 0.05), while *P* values > 0.05 are labeled ns (ns = no significance). *P* values < 0.01 is labeled with double asterisks (**P < 0.01), respectively. The mean of experimental groups beyond the confidence limits was considered statistically significant, but was labeled “ns” when the difference was within the confidence interval.

## Supplementary Information

**Additional file 1: Figure S1.** HIF-1α (hypoxia-inducible factor 1-alpha) protein levels are not affected by lactate treatment. Western blot analysis for relative protein expression. GAPDH (glyceraldehyde-3-phosphate dehydrogenase) was used as a loading control, with semi-quantification of western blot findings representing NDRG2 expression levels. One‐way ANOVA was used for statistical comparisons. Data are expressed as means ± SD. n = 3, ns = no significance, *P < 0.05 and **P < 0.05. **Figure S2.** *NDRG2*-silenced astrocytes were susceptible to apoptosis during oxygen–glucose deprivation (OGD) with lactate treatment. (A) Gene set enrichment analysis (GSEA) profiles for GSEA and signature sets. (B) Results of annexin-V-FITC/PI assay (fluorescein isothiocyanate-annexin V/propidium iodide) followed by flow cytometric quantification. Cells stained with annexin-V-FITC+/PI- are considered early apoptotic cells; cells stained with annexin-V-FITC+/PI+ are considered late apoptotic cells. **Figure S3.** Schematic of the *NDRG2* locus, wild-type (WT), the targeting vector, the targeted allele, and the deleted allele. The targeting vector replaces exons 2 and 6 with loxP-flanked exons 2 and 6, respectively. **Figure S4.** Comparison of siNDRG2 induced genes with human “MCAO (middle cerebral artery occlusion) induced” and “LPS (lipopolysaccharide) induced” specific genes identified by a previous study. The Venn diagram was generated using the “Draw Venn Diagram” website at http://bioinformatics.psb.ugent.be/webtools/Venn/ . **Figure S5.** Molecular docking pocket.

**Additional file 2: Table S1.** Classification of DEGs.

**Additional file 3: Table S2.** Hub genes identified by Cytoscape and their corresponding functions

**Additional file 4: Table S3.** Primers used in the qRT-PCR assay.

**Additional file 5: Table S4.** Differentially Expressed Transcripts.

## Data Availability

Any additional information required to reanalyze the data reported in this paper is available from the corresponding author with a completed Materials Transfer Agreement*.*

## Abbreviations

ANOVA: Analysis of variance
AP-1: Activator protein 1
C1q: Complement component 1q
CNS: Central nervous system
DEGs: Differentially expressed genes
ELISA: Enzyme-linked immunoadsorption assay
GAPDH: Glyceraldehyde-3-phosphate dehydrogenase
GFAP: Glial fibrillary acidic protein
H2-T23: Histocompatibility 2-T region locus 23
HE: Hematoxylin and eosin
HIF-1α: Hypoxia-inducible factor 1-alpha
IL1a: Interleukin-1a
JNK: C-Jun N-terminal kinase
LPS: Lipopolysaccharide
MAP2: Microtubule-associated protein 2
MCAO: Middle cerebral artery occlusion
MCT1: Monocarboxylate transporter 1
MG132: Carbobenzoxy-Leu-Leu-leucinal
NDRG: N-Myc downstream-regulated gene
OGD: Oxygen–glucose deprivation
qRT–PCR: Quantitative reverse transcription polymerase chain reaction
SD: Standard deviation
siRNA: Small inhibitory RNA
TAMs: Tumor-associated macrophages
TNF-R1: TNF type 1 receptor
TNF-R2: TNF type 2 receptor
TNFα: Tumor necrosis factor α
TRADD: TNFRSF1A-associated via death domain
TUNEL: Terminal deoxynucleotidyl transferase dUTP nick end labeling
V-FITC/PI: Fluorescein isothiocyanate-annexin V/propidium iodide

## References

[1] Jin R, Liu L, Zhang S, Nanda A, Li G (2013). Role of inflammation and its mediators in acute ischemic stroke. J Cardiovasc Transl Res.

[2] Jayaraj RL, Azimullah S, Beiram R, Jalal FY, Rosenberg GA (2019). Neuroinflammation: friend and foe for ischemic stroke. J Neuroinflammation.

[3] Giovannoni F, Quintana FJ (2020). The role of astrocytes in CNS inflammation. Trends Immunol.

[4] Sims NR, Yew WP (2017). Reactive astrogliosis in stroke: contributions of astrocytes to recovery of neurological function. Neurochem Int.

[5] Sofroniew MV, Vinters HV (2010). Astrocytes: biology and pathology. Acta Neuropathol.

[6] Rakers C, Schleif M, Blank N, Matuskova H, Ulas T, Handler K, Torres SV, Schumacher T, Tai K, Schultze JL (2019). Stroke target identification guided by astrocyte transcriptome analysis. Glia.

[7] Banerjee A, Ghatak S, Sikdar SK (2016). l-Lactate mediates neuroprotection against ischaemia by increasing TREK1 channel expression in rat hippocampal astrocytes in vitro. J Neurochem.

[8] Xu J, Zheng Y, Lv S, Kang J, Yu Y, Hou K, Li Y, Chi G (2020). Lactate promotes reactive astrogliosis and confers axon guidance potential to astrocytes under oxygen-glucose deprivation. Neuroscience.

[9] Bohn T, Rapp S, Luther N, Klein M, Bruehl TJ, Kojima N, Aranda Lopez P, Hahlbrock J, Muth S, Endo S (2018). Tumor immunoevasion via acidosis-dependent induction of regulatory tumor-associated macrophages. Nat Immunol.

[10] Liu N, Luo J, Kuang D, Xu S, Duan Y, Xia Y, Wei Z, Xie X, Yin B, Chen F (2019). Lactate inhibits ATP6V0d2 expression in tumor-associated macrophages to promote HIF-2alpha-mediated tumor progression. J Clin Invest.

[11] Lin K, Yin A, Yao L, Li Y (2015). N-myc downstream-regulated gene 2 in the nervous system: from expression pattern to function. Acta Biochim Biophys Sin.

[12] Flügge G, Araya-Callis C, Garea-Rodriguez E, Stadelmann-Nessler C, Fuchs E (2014). NDRG2 as a marker protein for brain astrocytes. Cell Tissue Res.

[13] Zhang Z, Ma Z, Zou W, Zhang L, Li Y, Zhang J, Liu M, Hou W, Ma Y (2019). N-myc downstream-regulated gene 2 controls astrocyte morphology via Rho-GTPase signaling. J Cell Physiol.

[14] Takarada-Iemata M, Kezuka D, Takeichi T, Ikawa M, Hattori T, Kitao Y, Hori O (2014). Deletion of N-myc downstream-regulated gene 2 attenuates reactive astrogliosis and inflammatory response in a mouse model of cortical stab injury. J Neurochem.

[15] Takarada-Iemata M, Yoshikawa A, Ta HM, Okitani N, Nishiuchi T, Aida Y, Kamide T, Hattori T, Ishii H, Tamatani T (2018). N-myc downstream-regulated gene 2 protects blood-brain barrier integrity following cerebral ischemia. Glia.

[16] Yin A, Guo H, Tao L, Cai G, Wang Y, Yao L, Xiong L, Zhang J, Li Y (2020). NDRG2 protects the brain from excitotoxicity by facilitating interstitial glutamate uptake. Transl Stroke Res.

[17] von Karstedt S (2018). NDRG2 programs tumor-associated macrophages for tumor support. Cell Death Dis.

[18] Li M, Lai X, Zhao Y, Zhang Y, Li M, Li D, Kong J, Zhang Y, Jing P, Li H (2018). Loss of NDRG2 in liver microenvironment inhibits cancer liver metastasis by regulating tumor associate macrophages polarization. Cell Death Dis.

[19] Wang L, Liu N, Yao L, Li F, Zhang J, Deng Y, Liu J, Ji S, Yang A, Han H (2008). NDRG2 is a new HIF-1 target gene necessary for hypoxia-induced apoptosis in A549 cells. Cell Physiol Biochem.

[20] Liu J, Zhang J, Wang X, Li Y, Chen Y, Li K, Zhang J, Yao L, Guo G (2010). HIF-1 and NDRG2 contribute to hypoxia-induced radioresistance of cervical cancer Hela cells. Exp Cell Res.

[21] Munoz Maniega S, Cvoro V, Chappell FM, Armitage PA, Marshall I, Bastin ME, Wardlaw JM (2008). Changes in NAA and lactate following ischemic stroke: a serial MR spectroscopic imaging study. Neurology.

[22] Li Y, Wang T, Zhang T, Lin Z, Li Y, Guo R, Zhao Y, Meng Z, Liu J, Yu X (2020). Fast high-resolution metabolic imaging of acute stroke with 3D magnetic resonance spectroscopy. Brain.

[23] Rehncrona S, Rosen I, Siesjo BK (1981). Brain lactic acidosis and ischemic cell damage: 1. Biochemistry and neurophysiology. J Cereb Blood Flow Metab.

[24] Hillered L, Hallstrom A, Segersvard S, Persson L, Ungerstedt U (1989). Dynamics of extracellular metabolites in the striatum after middle cerebral artery occlusion in the rat monitored by intracerebral microdialysis. J Cereb Blood Flow Metab.

[25] Harada K, Honmou O, Liu H, Bando M, Houkin K, Kocsis JD (2007). Magnetic resonance lactate and lipid signals in rat brain after middle cerebral artery occlusion model. Brain Res.

[26] Chaudhari P, Madaan A, Rivera JC, Charfi I, Habelrih T, Hou X, Nezhady M, Lodygensky G, Pineyro G, Muanza T, Chemtob S (2022). Neuronal GPR81 regulates developmental brain angiogenesis and promotes brain recovery after a hypoxic ischemic insult. J Cereb Blood Flow Metab.

[27] Hoque R, Farooq A, Ghani A, Gorelick F, Mehal WZ (2014). Lactate reduces liver and pancreatic injury in Toll-like receptor- and inflammasome-mediated inflammation via GPR81-mediated suppression of innate immunity. Gastroenterology.

[28] Zhai X, Li J, Li L, Sun Y, Zhang X, Xue Y, Lv J, Gao Y, Li S, Yan W (2020). L-lactate preconditioning promotes plasticity-related proteins expression and reduces neurological deficits by potentiating GPR81 signaling in rat traumatic brain injury model. Brain Res.

[29] Lauritzen KH, Morland C, Puchades M, Holm-Hansen S, Hagelin EM, Lauritzen F, Attramadal H, Storm-Mathisen J, Gjedde A, Bergersen LH (2014). Lactate receptor sites link neurotransmission, neurovascular coupling, and brain energy metabolism. Cereb Cortex.

[30] Lee DC, Sohn HA, Park ZY, Oh S, Kang YK, Lee KM, Kang M, Jang YJ, Yang SJ, Hong YK (2015). A lactate-induced response to hypoxia. Cell.

[31] Melotte V, Qu X, Ongenaert M, van Criekinge W, de Bruine AP, Baldwin HS, van Engeland M (2010). The N-myc downstream regulated gene (NDRG) family: diverse functions, multiple applications. FASEB J.

[32] Zhang D, Tang Z, Huang H, Zhou G, Cui C, Weng Y, Liu W, Kim S, Lee S, Perez-Neut M (2019). Metabolic regulation of gene expression by histone lactylation. Nature.

[33] Yu J, Chai P, Xie M, Ge S, Ruan J, Fan X, Jia R (2021). Histone lactylation drives oncogenesis by facilitating m(6)A reader protein YTHDF2 expression in ocular melanoma. Genome Biol.

[34] Hagihara H, Shoji H, Otabi H, Toyoda A, Katoh K, Namihira M, Miyakawa T (2021). Protein lactylation induced by neural excitation. Cell Rep.

[35] Guo H, Yin A, Ma Y, Fan Z, Tao L, Tang W, Ma Y, Hou W, Cai G, Zhuo L (2021). Astroglial N-myc downstream-regulated gene 2 protects the brain from cerebral edema induced by stroke. Glia.

[36] Liddelow SA, Guttenplan KA, Clarke LE, Bennett FC, Bohlen CJ, Schirmer L, Bennett ML, Munch AE, Chung WS, Peterson TC (2017). Neurotoxic reactive astrocytes are induced by activated microglia. Nature.

[37] Zamanian JL, Xu L, Foo LC, Nouri N, Zhou L, Giffard RG, Barres BA (2012). Genomic analysis of reactive astrogliosis. J Neurosci.

[38] Dembic Z, Loetscher H, Gubler U, Pan Y, Lahm H, Gentz R, Brockhaus M, Lesslauer W (1990). Two human TNF receptors have similar extracellular, but distinct intracellular, domain sequences. Cytokine.

[39] Vandenabeele P, Declercq W, Beyaert R, Fiers W (1995). Two tumour necrosis factor receptors: structure and function. Trends Cell Biol.

[40] Hsu H, Xiong J, Goeddel D (1995). The TNF receptor 1-associated protein TRADD signals cell death and NF-kappa B activation. Cell.

[41] Sedger LM, McDermott MF (2014). TNF and TNF-receptors: From mediators of cell death and inflammation to therapeutic giants - past, present and future. Cytokine Growth Factor Rev.

[42] Wang LW, Chang YC, Chen SJ, Tseng CH, Tu YF, Liao NS, Huang CC, Ho CJ (2014). TNFR1-JNK signaling is the shared pathway of neuroinflammation and neurovascular damage after LPS-sensitized hypoxic-ischemic injury in the immature brain. J Neuroinflammation.

[43] Maddahi A, Kruse LS, Chen QW, Edvinsson L (2011). The role of tumor necrosis factor-alpha and TNF-alpha receptors in cerebral arteries following cerebral ischemia in rat. J Neuroinflammation.

[44] Canedo T, Portugal CC, Socodato R, Almeida TO, Terceiro AF, Bravo J, Silva AI, Magalhaes JD, Guerra-Gomes S, Oliveira JF (2021). Astrocyte-derived TNF and glutamate critically modulate microglia activation by methamphetamine. Neuropsychopharmacology.

[45] Kim S, Son Y (2021). Astrocytes stimulate microglial proliferation and m2 polarization in vitro through crosstalk between astrocytes and microglia. Int J Mol Sci.

[46] Morris GM, Huey R, Lindstrom W, Sanner MF, Belew RK, Goodsell DS, Olson AJ (2009). Autodock4 and AutoDockTools4: automated docking with selective receptor flexiblity. J Comput Chem.

