# Supplementary material for: Lactate attenuates astrocytic inflammation by inhibiting ubiquitination and degradation of NDRG2 under oxygen–glucose deprivation conditions
Source: J Neuroinflammation. 2022 Dec 26;19:314. doi: 10.1186/s12974-022-02678-6 (PMC9793555; doi:10.1186/s12974-022-02678-6)
Supplement: Supplementary file 1 — Additional file 1: Figure S1. HIF-1α (hypoxia-inducible factor 1-alpha) protein levels are not affected by lactate treatment. Western blot analysis for relative protein expression. GAPDH (glyceraldehyde-3-phosphate dehydrogenase) was used as a loading control, with semi-quantification of western blot findings representing NDRG2 expression levels. One‐way ANOVA was used for statistical comparisons. Data are expressed as means ± SD. n = 3, ns = no significance, *P < 0.05 and **P < 0.05. Figure S2. NDRG2-silenced astrocytes were susceptible to apoptosis during oxygen–glucose deprivation (OGD) with lactate treatment. (A) Gene set enrichment analysis (GSEA) profiles for GSEA and signature sets. (B) Results of annexin-V-FITC/PI assay (fluorescein isothiocyanate-annexin V/propidium iodide) followed by flow cytometric quantification. Cells stained with annexin-V-FITC+/PI- are considered early apoptotic cells; cells stained with annexin-V-FITC+/PI+ are considered late apoptotic cells. Figure S3. Schematic of the NDRG2 locus, wild-type (WT), the targeting vector, the targeted allele, and the deleted allele. The targeting vector replaces exons 2 and 6 with loxP-flanked exons 2 and 6, respectively. Figure S4. Comparison of siNDRG2 induced genes with human “MCAO (middle cerebral artery occlusion) induced” and “LPS (lipopolysaccharide) induced” specific genes identified by a previous study. The Venn diagram was generated using the “Draw Venn Diagram” website at http://bioinformatics.psb.ugent.be/webtools/Venn/ . Figure S5. Molecular docking pocket. [file 12974_2022_2678_MOESM1_ESM.docx]

**Figures**

**
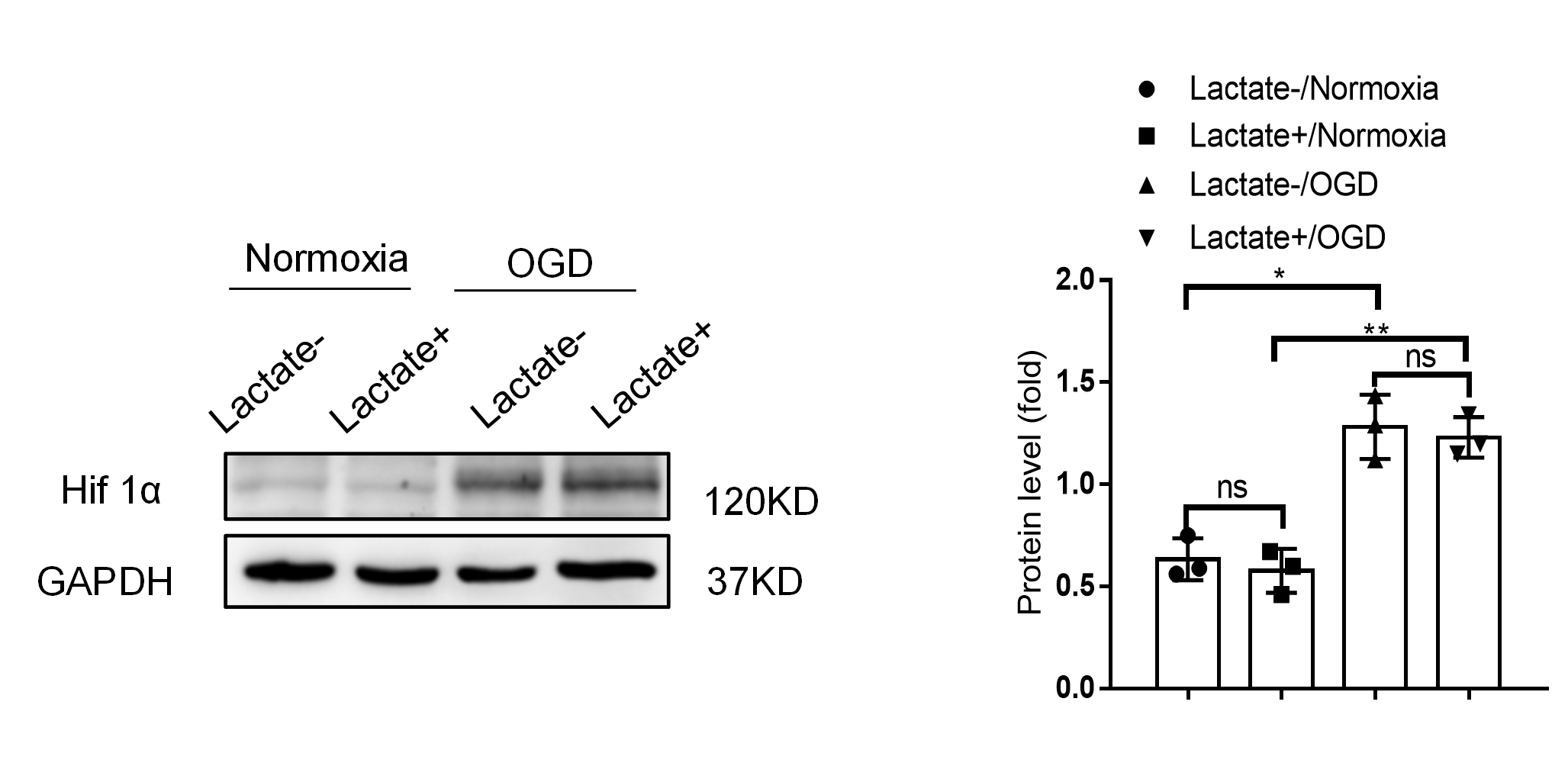
**

Figure S1. HIF-1α (hypoxia-inducible factor 1-alpha) protein levels are not affected by lactate treatment.

Western blot analysis for relative protein expression. GAPDH (glyceraldehyde-3-phosphate dehydrogenase) was used as a loading control, with semi-quantification of western blot findings representing NDRG2 expression levels. One‐way ANOVA was used for statistical comparisons. Data are expressed as means ± SD. n = 3, ns = no significance, **P* < 0.05 and ***P* < 0.05.


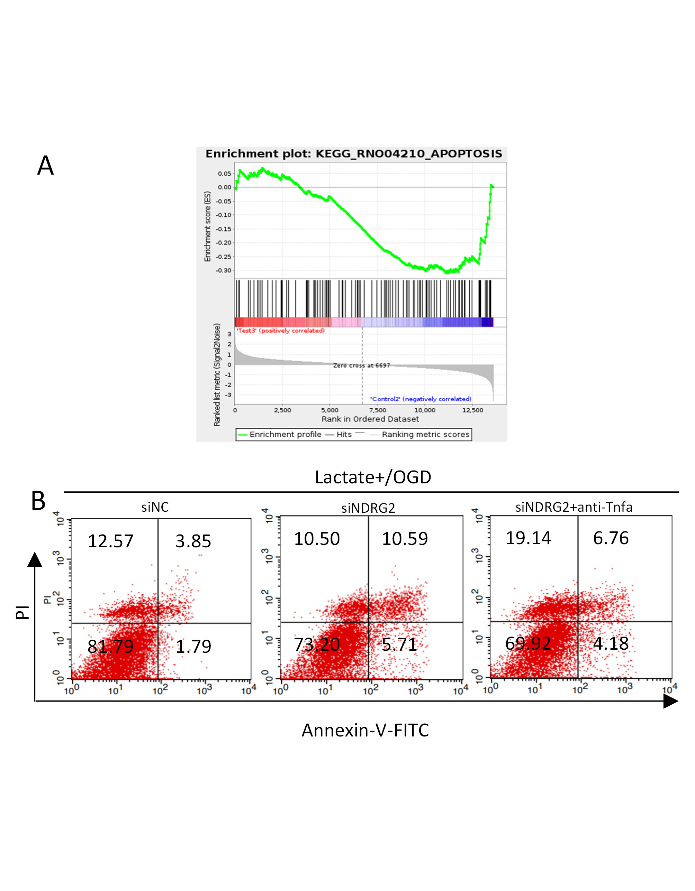


Figure S2. *NDRG2*-silenced astrocytes were susceptible to apoptosis during oxygen–glucose deprivation (OGD) with lactate treatment.

(A) Gene set enrichment analysis (GSEA) profiles for GSEA and signature sets. (B) Results of annexin-V-FITC/PI assay (fluorescein isothiocyanate-annexin V/propidium iodide) followed by flow cytometric quantification. Cells stained with annexin-V-FITC+/PI- are considered early apoptotic cells; cells stained with annexin-V-FITC+/PI+ are considered late apoptotic cells.


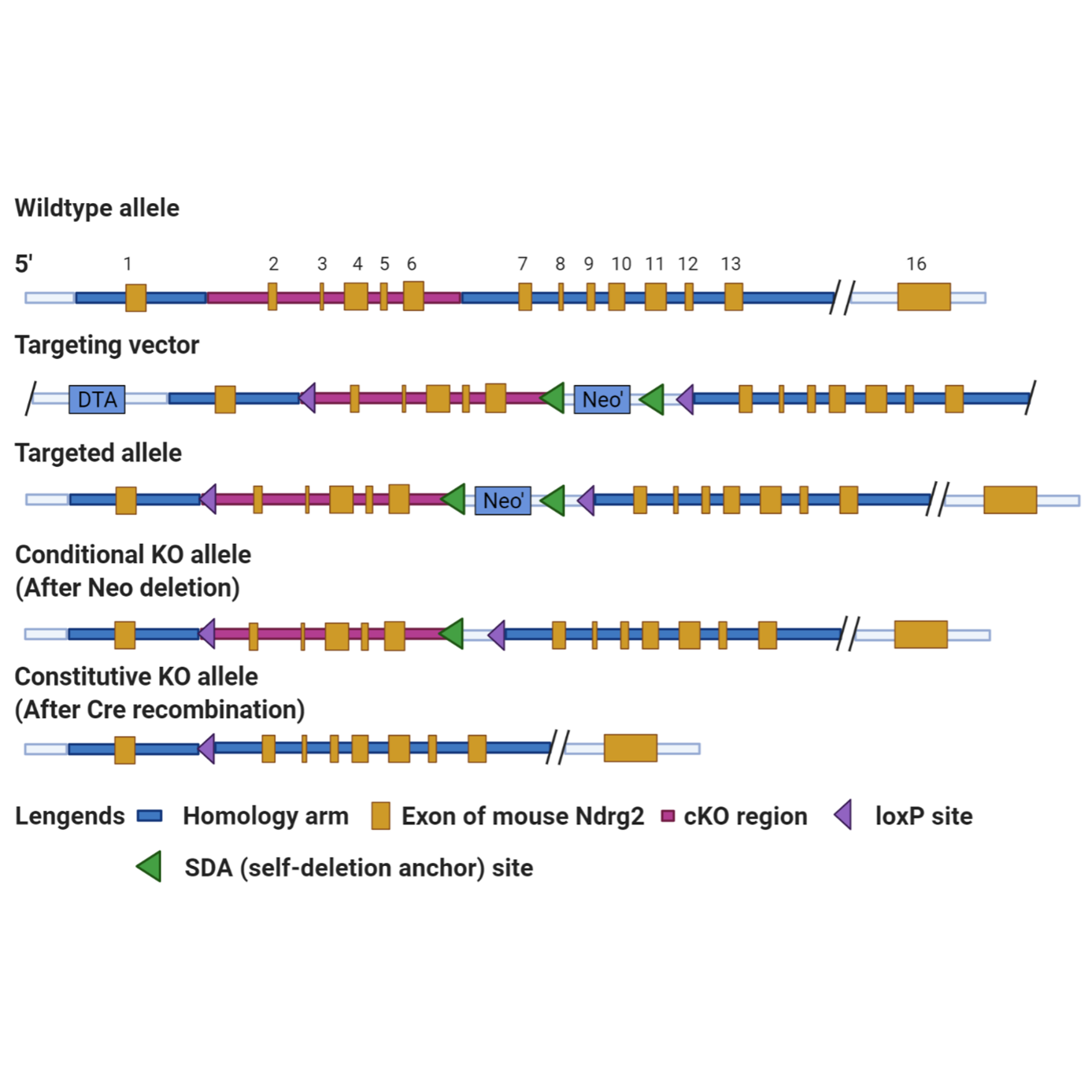


Figure S3. Schematic of the *NDRG2* locus, wild-type (WT), the targeting vector, the targeted allele, and the deleted allele

The targeting vector replaces exons 2 and 6 with loxP-flanked exons 2 and 6, respectively.


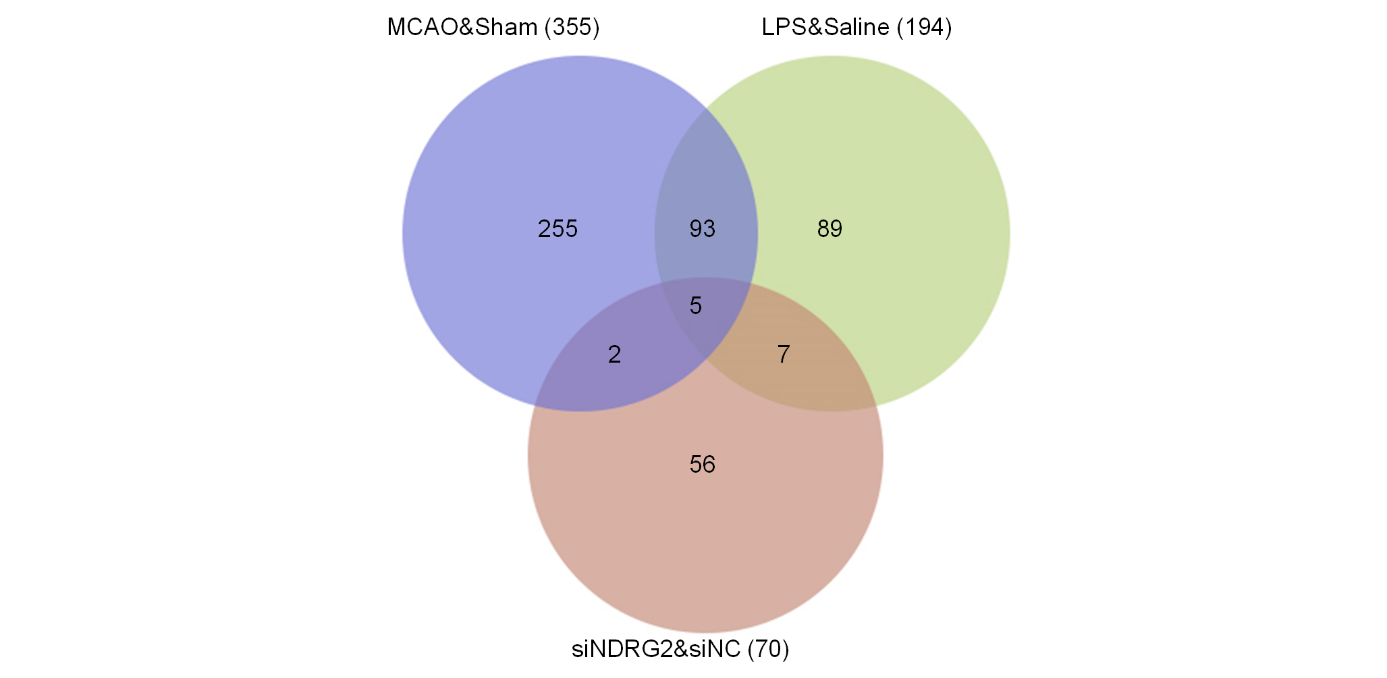


Figure S4. Comparison of siNDRG2 induced genes with human “MCAO (middle cerebral artery occlusion) induced” and “LPS (lipopolysaccharide) induced” specific genes identified by a previous study

The Venn diagram was generated using the “Draw Venn Diagram” website at <http://bioinformatics.psb.ugent.be/webtools/Venn/> .


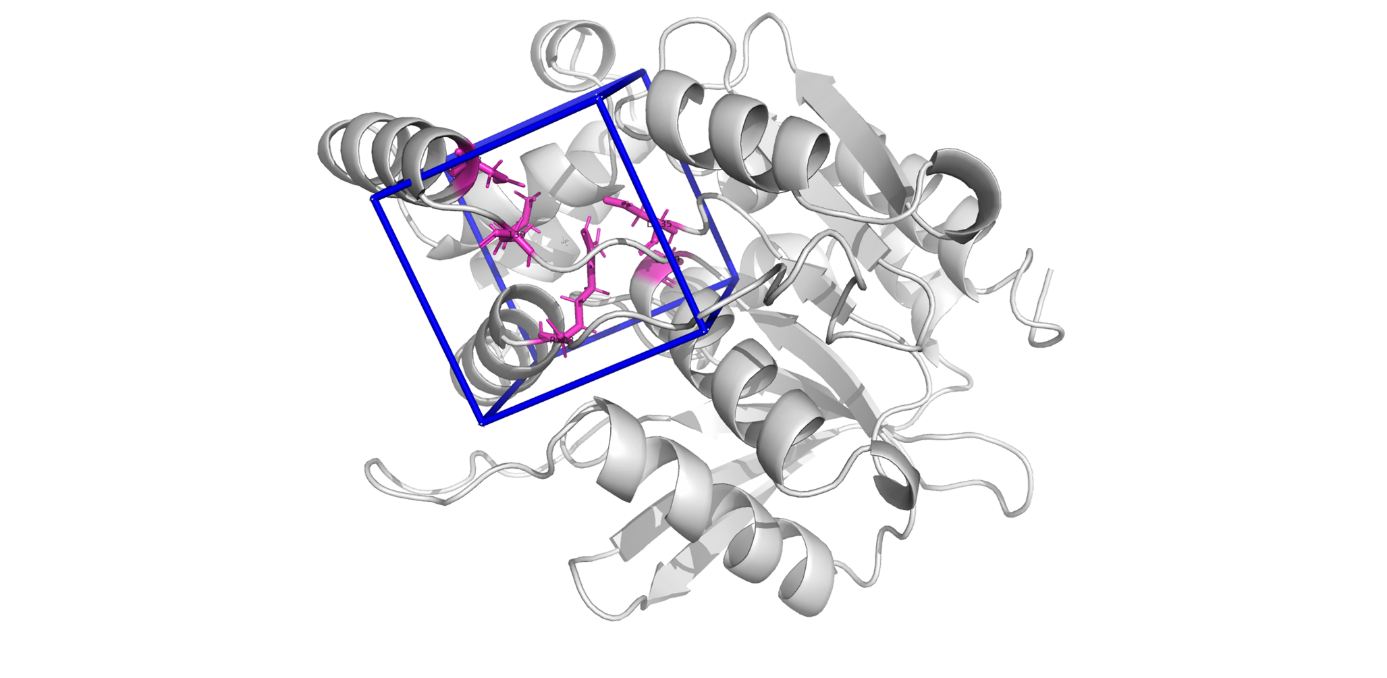


**Figure S5. Molecular docking pocket**
