# Supplementary material for: Lactate attenuates astrocytic inflammation by inhibiting ubiquitination and degradation of NDRG2 under oxygen–glucose deprivation conditions
Source: J Neuroinflammation. 2022 Dec 26;19:314. doi: 10.1186/s12974-022-02678-6 (PMC9793555; doi:10.1186/s12974-022-02678-6)
Supplement: Supplementary file 3 — Additional file 3: Table S2. Hub genes identified by Cytoscape and their corresponding functions [file 12974_2022_2678_MOESM3_ESM.docx]

**Table S2.** Hub genes identified by Cytoscape and their corresponding functions

| Gene | Aliases | Function |
| --- | --- | --- |
| STAT1 | Signal Transducer and Activator of Transcription 1 | DNA-binding transcription factor activity; protein homodimerization activity; signal transducer and transcription activator mediating cellular responses to interferons (IFNs); cytokine KITLG/SCF, other cytokines; other growth factors |
| CXCL10 | C-X-C Motif Chemokine Ligand 10 | Signaling receptor binding and chemokine activity; pro-inflammatory cytokines involved in a wide variety of processes, such as chemotaxis, differentiation, and activation of peripheral immune cells; regulation of cell growth; apoptosis; modulation of angiostatic effects |
| IRF7 | Interferon Regulatory Factor 7 | DNA-binding transcription factor activity; key transcriptional regulator of type I interferon (IFN)-dependent immune responses; innate immune response against DNA and RNA viruses |
| TNFα | Tumor Necrosis Factor α (belongs to the TNF superfamily) | Identical protein binding; cytokine activity; pro-inflammatory cytokine |
| SPP1 | Secreted Phosphoprotein 1 | Cytokine activity; extracellular matrix binding; cytokine involved in enhancing the production of interferon-gamma and interleukin-12 and reducing the production of interleukin-10; type I immunity pathway |
| CD44 | CD44 Molecule | Transmembrane signaling receptor activity; cytokine receptor activity |
| C3 | Complement C3 | Activation of the complement system; signaling receptor binding; C5L2 anaphylatoxin chemotactic receptor binding; A1-like astrocyte marker genes |
| RT1-S3 | RT1 class 1b, locus S3 | Signaling receptor binding; MHC class I protein binding; A1-like astrocyte marker gene; identical protein binding |
| B2M | Beta-2-Microglobulin | Identical protein binding; diseases associated with B2M; immunodeficiency 43; amyloidosis, familial visceral |
| PSMB8 | Proteasome 20S Subunit Beta 8 | proteasome-associated autoinflammatory syndrome 1; proteasome-associated autoinflammatory syndrome |
