# Supplementary material for: Lactate attenuates astrocytic inflammation by inhibiting ubiquitination and degradation of NDRG2 under oxygen–glucose deprivation conditions
Source: J Neuroinflammation. 2022 Dec 26;19:314. doi: 10.1186/s12974-022-02678-6 (PMC9793555; doi:10.1186/s12974-022-02678-6)
Supplement: Supplementary file 4 — Additional file 4: Table S3. Primers used in the qRT-PCR assay. [file 12974_2022_2678_MOESM4_ESM.docx]

**Table S3. Primers used in the qRT-PCR assay**

| Gene | Primer sequence | Fragment length |
| --- | --- | --- |
| Gapdh qPCR Forward (rat) | AGACAGCCGCATCTTCTTGT | 207 |
| Gapdh qPCR Reverse (rat) | CTTGCCGTGGGTAGAGTCAT |  |
| Tnfα qPCR Forward (rat) | AAATGGGCTCCCTCTCATCAGTTC | 180 |
| Tnfα qPCR Reverse (rat) | TCCGCTTGGTGGTTTGCTACGAC |  |
| Ndrg2 qPCR Forward (rat) | CCCACACAGACCTCATTCCT | 141 |
| Ndrg2 qPCR Reverse (rat) | AGACAGGCGAGTCATACAGG |  |
| Cxcl10 qPCR Forward (rat) | TCCTGCAAGTCTATCCTGTC | 159 |
| Cxcl10 qPCR Reverse (rat) | TGGCTTCTCTCTAGTTACGG |  |
| Cxcl11 qPCR Forward (rat) | CGAAGAAAGATCACCAGAGCCA | 132 |
| Cxcl11 qPCR Reverse (rat) | CCCCCTTTGAACATAACGAAGC |  |
| Ifnβ1 qPCR Forward (rat) | TTGCGTTCCTGCTGTGCTTCTC | 142 |
| Ifnβ1 qPCR Reverse (rat) | TCCGTCCTGTAGCTGAGGTTGAG |  |
| Il12α qPCR Forward (rat) | CTGAATCACAGCGGCGAGACTC | 99 |
| Il12α qPCR Reverse (rat) | GAAGGCATGGAGCAGGATACAGAG |  |
| Ripk3 qPCR Forward (rat) | CAAAAAGGTACAGAGGTGGATTGC | 61 |
| Ripk3 qPCR Reverse (rat) | GGCGGTCCAGCATTTCAT |  |
| Pik3r1 qPCR Forward (rat) | CGAAAACACAGAAGACCAATACTCA | 122 |
| Pik3r1 qPCR Reverse (rat) | TCCCTCGCAATAGGTTCTCG |  |
| Spp1 qPCR Forward (rat) | CCGAGGTGATAGCTTGGCTT | 123 |
| Spp1 qPCR Reverse (rat) | TCGGACTCCTGGCTCTTCAT |  |
| Stat1 qPCR Forward (rat) | GAACGTGCTCTGCTCAAGGA | 130 |
| Stat1 qPCR Reverse (rat) | GAACGTGCTCTGCTCAAGGA |  |
| Irf7 qPCR Forward (rat) | CCCTCAGCAGTGCCCTACCC | 107 |
| Irf7 qPCR Reverse (rat) | CCCAGCATCACCAGAAAGCAGAG |  |
| C3 qPCR Forward (rat) | TCGAAATCCCTCCCAAGTC | 60 |
| C3 qPCR Reverse (rat) | CGATCTTCAAGGGGACAATG |  |
| Tradd qPCR Forward (rat) | TGGCAATCTACAAGGCTCTG | 116 |
| Tradd qPCR Reverse (rat) | GAAACGCAACTGAACGATGA |  |
| Tnfrsfα qPCR Forward (rat) | GCTGTTGCCTCTGGTTATCT | 146 |
| Tnfrsf1α qPCR Reverse (rat) | GCTGTTGCCTCTGGTTATCT |  |
| Actin qPCR Forward (mouse) | CGTGGGCCGCCCTAGGCACCA | 243 |
| Actin qPCR Reverse (mouse) | TTGGCCTTAGGGTTCAGGGGGG |  |
| Tnfα qPCR Forward (mouse) | CTGTAGCCCACGTCGTAGC | 97 |
| Tnfα qPCR Reverse (mouse) | TTGAGATCCATGCCGTTG |  |
| Neo-del qPCR Forward (mouse) | AGGACCTCTGGCATCTTAAGTAGT | __ |
| Neo-del qPCR Reverse (mouse) | TGGACCTTAAAGCAACGTATGAAAC |  |
| loxP qPCR Forward (mouse) | TTTTAGCCCCTCAAGATGCTGAC | __ |
| loxP qPCR Reverse (mouse) | TCACCTCGTACCTCACATGC |  |
| GFAP-Cre qPCR Forward (mouse) | TAGCCCACTCCTTCATAAAGCCCT | __ |
| GFAP-Cre qPCR Reverse (mouse) | GCTAAGTGCCTTCTCTACACC |  |
